# Fluoroquinolone-related adverse events resulting in health service use and costs: A systematic review

**DOI:** 10.1371/journal.pone.0216029

**Published:** 2019-04-26

**Authors:** Laura S. M. Kuula, Kati M. Viljemaa, Janne T. Backman, Marja Blom

**Affiliations:** 1 Faculty of Pharmacy, University of Helsinki, Helsinki, Finland; 2 Individualized Drug Therapy Research Program, Faculty of Medicine, University of Helsinki, Helsinki, Finland; 3 Department of Clinical Pharmacology, University of Helsinki, Helsinki, Finland; 4 Helsinki University Hospital, Helsinki, Finland; University of Maryland School of Medicine, UNITED STATES

## Abstract

**Background and objectives:**

Adverse events (AEs) associated with the use of fluoroquinolone antimicrobials include *Clostridium difficile* associated diarrhea (CDAD), liver injury and seizures. Yet, the economic impact of these AEs is seldom acknowledged. The aim of this review was to identify health service use and subsequent costs associated with ciprofloxacin, levofloxacin, moxifloxacin, norfloxacin and ofloxacin -related AEs.

**Methods:**

A literature search covering Medline, SCOPUS, Cinahl, Web of Science and Cochrane Library was performed in April 2017. Two independent reviewers systematically extracted the data and assessed the quality of the included studies. All costs were converted to 2016 euro in order to improve comparability.

**Results:**

Of the 5,687 references found in the literature search, 19 observational studies, of which five were case-controlled, fulfilled the inclusion criteria. Hospitalization was an AE-related health service use outcome in 17 studies. Length of hospital stay associated with AEs varied between <5 and 45 days. The estimated cost of an AE episode ranged between 140 and 18,252 €. CDAD was associated with the longest stays in hospital. Ten studies reported AE-related length of stays and five evaluated costs associated with AEs. Due to the lack of published literature, health service use and costs associated with many high-risk FQ-related AEs could not be evaluated.

**Conclusions:**

Because of the wide clinical use of fluoroquinolones, in particular serious fluoroquinolone-related AEs can have substantial economic implications, in addition to imposing potentially devastating health complications for patients. Further measures are required to prevent and reduce health service use and costs associated with fluoroquinolone-related AEs. Equally, better-quality reporting and additional published data on health service use and costs associated with AEs are needed.

## Introduction

Fluoroquinolones (FQs) are counted among broad-spectrum antimicrobials and are used to treat genitourinary, respiratory, gastrointestinal, skin and soft tissue infections[1]. FQs are generally well tolerated antimicrobials: the discontinuation of treatment due to AEs is required in fewer than five percent of consumption[2]. Their mechanism of action is based on the drugs’ ability to inhibit DNA gyrase and topoisomerase IV, and thus, DNA synthesis[3]. The most common AEs are mild and reversible, such as diarrhea, nausea and headaches. However, FQs are also associated with more serious AEs, including *Clostridium difficile* infections, prolonged QT interval, tendinitis and tendon rupture, dysglycemia, hepatic toxicity, phototoxicity, acute renal failure and serious AEs involving the central nervous system, such as seizures. [4] [1] FQ-related AEs can be multisymptomatic, progressive and have long latency periods, which can make them difficult to detect[5]. FQs have been in clinical use since the 1980s[6] and are globally among the most consumed antimicrobials[7]. Due to reported serious AEs associated with the use of FQs, the European Medicines Agency (EMA) recommended restrictions on their use in October 2018.[8] The U.S. Food and Drugs Administration (FDA) has issued several “black box warnings” against FQs with the latest safety announcement dated in December 2018 warning about an increased risk of ruptures or tears in the aorta blood vessel in some patients.[9] FDA-approved FQs are ciprofloxacin, levofloxacin, moxifloxacin, ofloxacin, gemifloxacin and recently, delafloxacin[10][11]. FQs approved in Europe include ciprofloxacin, levofloxacin, moxifloxacin, ofloxacin, gemifloxacin, cinoxacin, enoxacin, flumequine, lomefloxacin, nalidixic acid, norfloxacin, pefloxacin, pipemidic acid, prulifloxacin and rufloxacin.

The economic burden of AEs is substantial and in direct relation to current increasing drug utilization. According to previous research, the annual cost of AEs in the U.S. may be as high as 22.9 billion euros [12]. In Europe AEs are considered to contribute to 3.6 percent of hospital admissions, have an impact on 10 percent of inpatients during their hospital admission and are responsible for almost 0.5 percent of inpatient deaths. [13] AEs thus clearly constitute a major clinical issue. Prescribing a drug is always a conflict of benefits set against harms decision, weighing the risk of morbidity and even mortality from the disease against similar effects from AEs and added health care costs. Unfortunately, a thorough understanding of the significance of AEs and the benefit-risk-ratio of drug treatments can only be acquired through long-term clinical use after marketing authorization and subsequent research. Health service use and costs specifically associated with FQ-related AEs have not been evaluated previously.

The aim of our study was to identify health service use and health service costs associated with ciprofloxacin, levofloxacin, moxifloxacin, norfloxacin and ofloxacin -related AEs.

## Methods

### Literature search

A systematic literature search was performed in April 2017 covering Medline, SCOPUS, CINAHL, Web of Science and Cochrane Library. A library information specialist was consulted in forming the search strategies, which consisted of search terms relating to FQs, AEs, health service use and costs. The Web of Science -database search included several conference papers, which could be used to find unpublished literature and reduce publication bias. Finally, literature references of the included articles were sourced to identify potentially relevant articles. The search strategy for Medline can be found in S1 File. In this systematic review, AEs are defined as medical occurrences temporally associated with the use of a medicinal product, but not necessarily causally related. A serious adverse event, on the other hand, is defined as any untoward medical occurrence that at any dose either results in death, is life threatening, requires inpatient hospitalization or prolongation of existing hospitalization or results in persistent or significant disability or incapacity. [14] Health service use is referred to as services provided to individuals or communities by health service providers for the purpose of promoting, maintaining, monitoring or restoring health[15]. Costs presented in this study comprise resources consumed due to health service use.

### Study selection

References identified in the literature search were imported to reference management software (Mendeley) and duplicates were removed. Only references that met previously fixed PICOS (patients, intervention, control, outcome, setting) [16] criteria, were included in the review. There were no limitations concerning publication year. The PICOS framework is depicted in Table 1.

**Table 1:** Inclusion and exclusion criteria.

	Inclusion criteria	Exclusion criteria
Patient	Adults (≥ 16-year old patients)	Children (< 16-year old patients) Animals
Intervention	Single systemic use of levo-, cipro-, moxi-, nor-, or ofloxacin	Other intervention, FQ as a part of combination therapy or not systemic use
Comparison	Other intervention, placebo, no comparison	-
Outcome	Levo-, cipro-, moxi-, nor-, oflo-related AE resulting in health service use and/or costs	No reported levo-, cipro-, moxi-, nor-, oflo-related AE health service use and/or costs
Study design	RCT, observational studies	Case reports, case series Published only as abstract No English full-text

Both reviewers (LK, KV) individually screened the articles based on title and excluded distinctly irrelevant references such as literature regarding topical ophthalmic FQs. A third author (MB) was available to resolve possible discrepancies. The remaining articles were screened based on abstracts and full texts. The number of identified, included and excluded references are depicted in the flow chart.

### Data collection

The data of the included articles was extracted into two spread sheets (Microsoft Excel). The usefulness of the tables was tested with a total of eight articles, after which minor adjustments were made regarding the reporting of fatalities. Both reviewers (LK, KV) filled in both tables independently. The first table contains characteristics of the included studies, such as authors, publication years, aims, patient details, study designs, durations, follow-ups, funding details and publications. The second table summarizes results covering specifics of the fluoroquinolone associated with the adverse event, adverse event types, health service use, length of hospital stay, AE costs and possible fatalities. In order to improve comparability, all the reported costs were converted to euro by using the exchange rate of the European Central Bank and adjusted to the price level of the year 2016 using the value of money index of Statistics Finland[17][18].

### Quality assessment

The quality of the included studies was assessed according to the STROBE checklist for observational studies.[19] The studies were awarded scores, which are presented in percentages. Two reviewers (LK, KV) assessed the quality of the included studies independently. The level of agreement between the reviewers was 93%.

## Results

### Search results

**Fig 1:**
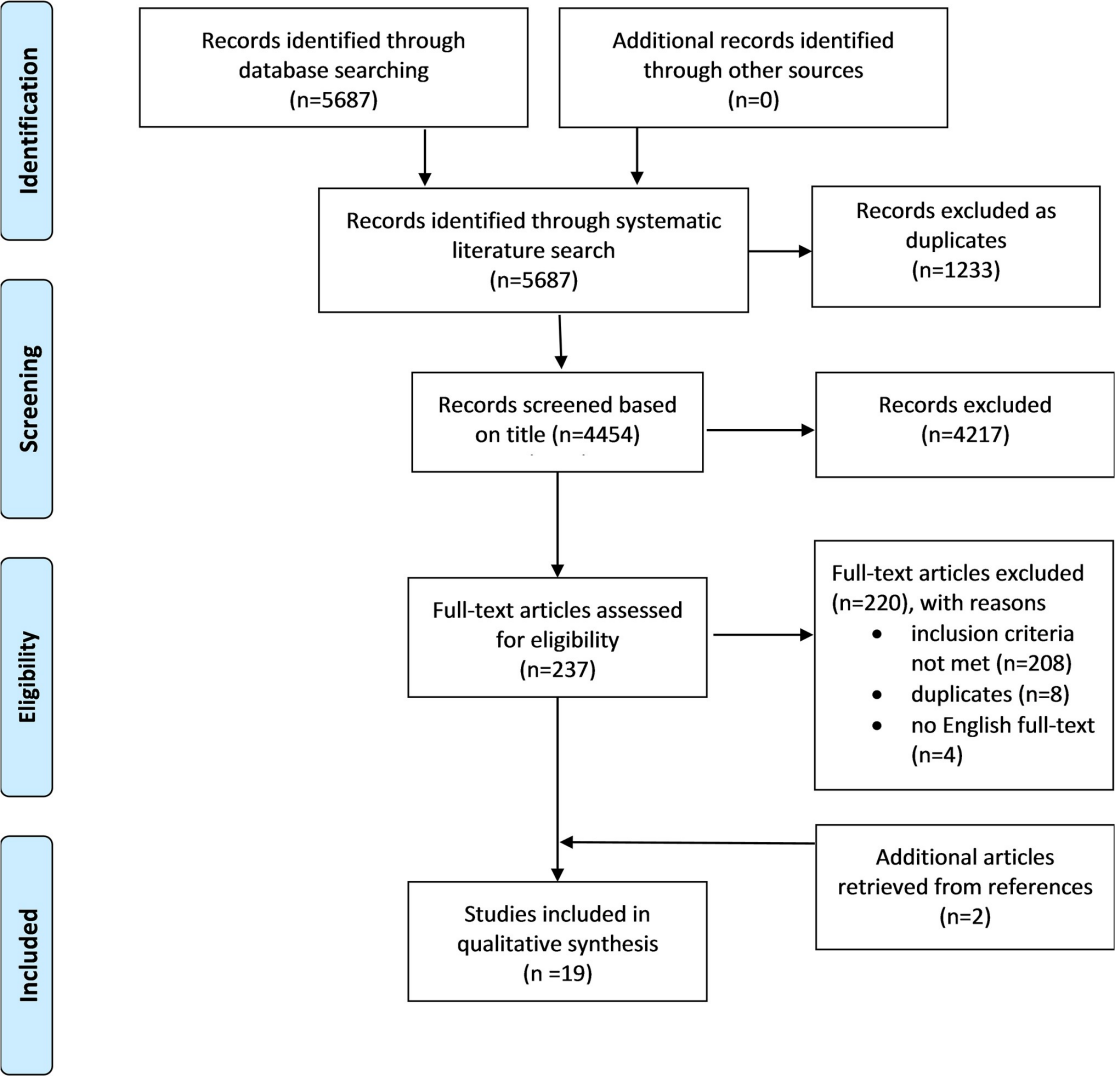
Flow chart of the study selection process.

In all, 4,454 unique references were identified in the literature search (Fig 1). Screening based on titles excluded 4,217 references. Two hundred and twenty full-text articles did either not meet the inclusion criteria (n = 208 studies), were found to be duplicates (n = 8) or lacked an English language full-text (n = 4). After two additional studies were found in literature references, a total of 19 studies were included in this systematic review. The list of the excluded articles is displayed in S2 File.

### Study characteristics

Of the 19 included observational studies ([20]-[31]), five were case-controlled ([20][21][22][23][24]). The studies were published between 2002 and 2017. There were substantial differences in study duration, the length varied from 4 weeks to 22 years. The total sample size of the included studies comprised 1,752,544 patients. During the study periods, 33,477 AEs that were identified as FQ-related occurred. The studies included 22,704 AEs associated with levofloxacin, 339 with ciprofloxacin, two with norfloxacin, three with ofloxacin and 168 with moxifloxacin. In total, 10,773 AEs were associated with an unspecified FQ. A total of 26,893 (80%) were identified from one study[25]. The average age of all total sample was 60,8 years and 50,71% were men. Only one study explicitly involved a cohort of patients with comorbidities (diabetes).[26] The characteristics of the included studies are summarized in Table 2.

**Table 2:** Characteristics of the studies (n = 19) included in the current review.

Study, Year of publication, Country	Aim of the Study	Patients	Study design	Study duration	Follow-up to AE	Source of research funding	Journal
**Case-controlled studies**							
Dhalla et al. 2006, Canada [20]	To determine if community-acquired CDAD was more strongly associated with gati and moxi than with levo	Cases: Patients (n = 96, mean age, years 80, IQR 76–84, male sex 44.8%) with a prescription for levo, moxi, gati or cipro admitted to hospital with a diagnosis of CDAD. Controls: Patients with a prescription for levo, cipro, gati or moxi with no hospitalization involving CDAD (n = 941, mean age, years 80, IQR 75–83, male sex 44.3%)	Population-based, nested case-control study	3 years	30 days	Canadian Institutes for Health Research	Antimicrobial Agents and Chemotherapy
Kaye et al. 2014, USA [21]	To estimate the incidence and relative risk of a hospitalization or emergency visit for noninfectious liver injury in users of eight oral antimicrobials—amoxicillin, amoxicillin-clavulanic acid clarithromycin, cefuroxime, doxycycline, levo, moxi, telithromycin—compared with nonusers of these antimicrobials	Cases: Patients (n = 607, mean age, years 56.5, male sex 45%) with or without antimicrobial exposure and subsequent diagnosis indicating noninfectious liver injury. Controls: Patients (n = 6070, mean age, years 56.1, male sex 45%) with or without antimicrobial exposure without subsequent diagnosis indicating noninfectious liver injury.	Retrospective observational cohort study with nested case-control analysis	7 years 9 months	30 days and 90 days	Bayer Pharma AG	Pharmacotherapy
McFarland et al. 2007, USA [22]	To test the hypothesis that the increase in CDAD incidence was associated with the formulary change of replacing levo with gati, and to determine CDAD risk factors for the study population	Cases: Inpatients (n = 164, mean age, years ± SD 65.9 (13.4), male sex NR) and outpatients (n = 20, mean age, years ± SD 56.5 (48.5), male sex NR) with CDAD. Controls: inpatients and outpatients without CDAD (n = 184, mean age, years ± SD NR, male sex NR)	Retrospective, matched case-control study	Unclear	3 months	The Seattle Epidemiologic Research and Information Center	Clinical Infectious Diseases
Muto et al. 2005, USA [23]	To identify risk factors for Clostridium difficile acquisition and characterize the outbreak	Cases: Patients admitted to hospital with CDAD (n = 203, median age, years (range) 64 (17–95), male sex 51.2%) Controls: Patients admitted to hospital without CDAD (n = 203, median age, years (range) 59 (16–93), male sex 52.2%)	Retrospective case-control study	1 year 4 months	28 days	The National Institute of Allergy and Infectious Diseases	Infection Control and Hospital Epidemiology
Paterson et al. 2012, Canada [24]	To explore the association of FQ use with subsequent admission to hospital for acute hepatotoxicity	Cases: Patients with no history of liver disease admitted to hospital with acute liver injury, prior use of broad-spectrum antibiotics (n = 144, mean age, years ± SD 77.4 (7.9), male sex 52.8%) Controls: Patients with no acute liver injury subsequent to broad-spectrum antibiotic use (n = 1409, mean age, years ± SD 77.0 (7.5), male sex 52.4%)	Population-based, nested, case-control study	9 years	30 days	The Canadian Institutes of Health Research and The Institute for Clinical Evaluative Sciences	Canadian Medical Association Journal
**Cohort studies**							
Aspinall et al. 2009, USA [27]	To compare the risk of severe hypo- and hyperglycemia in a cohort of patients treated with gati, cipro, or levo or with a non-FQ antibiotic, azithromycin	Outpatients with a prescription for gati (n = 218,748, mean age ± SD, years 62.9 (13.8), male sex 93.7%), levo (n = 457,994, mean age ± SD, years 63.5 (13.5) male sex 94.2%), cipro (n = 197,940, mean age ± SD, years 62.8 (13.6), male sex 93.7%) or azithromycin (n = 402,566, mean age ± SD, years 58.2 (14.7), male sex 89.5%)	Retrospective inception cohort study	5 years	15 days	The Veterans Affairs Center for Medication Safety	Clinical Infectious Diseases
Chou et al. 2013, Taiwan [26]	To assess the risk of severe dysglycemia among diabetic patients who received different FQ	Diabetic patients with new prescriptions for oral cipro (n = 12,564, mean age ± SD, years 66.4 (13.2), male sex 42.2%), levo (n = 11,766, mean age ± SD, years 67.0 (12.8), male sex 48.4%), moxi (n = 4,221, mean age ± SD, years 67.6 (13.0), male sex 57.1%), second-generation cephalosporins (n = 20,317, mean age ± SD, years 62.4 (14.2), male sex 41.7%) or macrolides (n = 29,565, mean age ± SD, years 62.4 (12.6), male sex 52.0%)	Population-based inception cohort study	1 year 11 months	30 days	The Taiwan Department of Health	Clinical Infectious Diseases
Mah et al. 2011, USA [28]	To examine how age and levo exposure influence the absolute risk of CDI in an academic medical center	Patients exposed to levo (n = 2,636, age 20–99 years, male sex % NR) or ceftriaxone (n = 1,267, age 20–99 years, male sex % NR)	Retrospective cohort study	2 years	30 days	No funding to disclose	Infectious Diseases in Clinical Practice
Perrone et al. 2014, Italy [29]	To determine the prevalence, preventability, seriousness requiring hospitalization, subsequent 30-day mortality, and economic impact of ADRs presenting to multiple EDs serving a large proportion of the Lombardy region over a 2-year period	Patients (n = 8,862) presenting to the ED with ADR (mean age, years± SD 55.9 (24.3), male sex 44.3%)	Retrospective cohort study	2 years	NA	Regional Pharmacovigilance Centre of Lombardy, Italian Medicines Agency (AIFA)	Clinicoeconomics and Outcomes Research
**Other prospective and retrospective observational studies**							
Jamunarani and Priya 2014, India [30]	To see the clinical spectrum of ADR related hospital admissions in a tertiary care hospital, to establish a causal link between the drug and reaction, and to identify common challenges encountered in ADR collection process and methods to promote ADR reporting	Patients hospitalized due to ADRs (n = 33, mean age NR, male sex 45.5%)	Cross sectional analytical study	1 year 1 month	NA	NR	Asian Journal of Pharmaceutical and Clinical Research
Llop et al. 2017, USA* [25]	To investigate real-world outcomes and costs associated with the use of current guideline-recommended antimicrobial treatments for CAP in both the outpatient and inpatient settings	Outpatients (n = 165,768, age, years 53.1 ± 16.4, male sex 51.0%) diagnosed with CAP and treated with FQ, macrolide (n = 169,335, age, years 47.4 ± 16.8, male sex 48.0%) or beta-lactam (n = 36,702, age, years 51.7 ± 18.1, male sex 49.1%)	Claims-based retrospective study	6 years	30 days	Cempra Pharmaceuticals	Hospital Practice
Martí et al. 2005, Spain [31]	To ascertain the epidemiological characteristics, clinical symptoms, and evolution of drug-induced hepatitis over 22 years	Inpatients and outpatients with a diagnosis of drug-induced hepatitis (n = 61, mean age, years ± SD 52.4 (17), male sex 42.6%)	Retrospective observational study and prospective study	22 years	NA	NR	Revista Española de Enfermedades Digestivas
Mjörndal et al. 2002, Sweden [32]	To determine the occurrence and pattern of ADRs as a cause for acute admission into a clinic of internal medicine	Patients (n = 82, median age, years (range) 74 (21–92), male sex 46.3%) admitted to hospital due to ADR compared with patients (n = 587, median age, years (range) 72 (19–97), male sex 49.1%) admitted to hospital due to other causes	Prospective study	36 weeks	NA	The Federation of Swedish County Councils	Pharmacoepidemiology and Drug Safety
Noel et al. 2004, India [33]	To evaluate the clinical spectrum of all cutaneous ADRs over one year in hospitalized patients in the Department of Dermatology and the establish the causal link between the suspected drug and the reaction by using the WHO causality definitions	Patients admitted to the Department of Dermatology diagnosed with cutaneous ADRs (n = 56, mean age unclear, male sex 50%)	Prospective hospital-based study	1 year	NA	NR	Indian Journal of Pharmacology
Olivier et al. 2002, France [34]	To assess the incidence and the preventability of ADR-related admissions and to assess the feasibility of a wider use of a preventability scale in clinical practice	Patients presenting to an ED with a suspected ADR (n = 41, mean age, years ± SD 58 (22.2), male sex 54%) compared with patients presenting to an ED for other reasons than suspected ADR (n = 630, mean age, years ± SD 55.6 (22.5), male sex 55%)	Prospective pharmacovigilance study	4 weeks	NA	No funding to disclose	Drug Safety
Patel et al. 2007, India [35]	To evaluate the prevalence of patients presenting with ADRs to the ED and to assess the causality, avoidability, and severity of ADRs. The study also aimed to determine the economic burden of ADRs from a hospital perspective.	Patients (n = 265, mean age, years 40, male sex % unclear) admitted to ED due to ADRs.	Prospective observational study	6 weeks	NA	NR	BMC Clinical Pharmacology
Sánchez Muñoz-Torrero et al. 2010, Spain [36]	To assess the prevalence of ADRs in the internal medicine wards of two teaching hospitals, identify the most common ADRs, the principal medications involved, and determine the risk factors implicated in the occurrence of such ADRs	Patients admitted to hospital with ADRs (n = 126, median age, years (range) 69 (16–97), male sex 47%) compared with patients admitted to hospital without ADRs (n = 279, median age, years (range) 67 (15–102), male sex 54%)	Prospective observational study	10 weeks	NA	NR	European Journal of Clinical Pharmacology
Su and Aw 2014, Singapore [37]	To look at the epidemiology of SCAR in the local setting in Singapore and the underlying characteristics of our patients that may influence the drug reaction seen	Inpatients (n = 42), mean age 51.8 years, male sex 50%	Retrospective study	5 years	NA	NR	International Journal of Dermatology

AE, adverse event; ADE, adverse drug event; ADR, adverse drug reaction; CAP, community-acquired pneumonia; CDAD, Clostridium difficile-associated disease; CDI, clostridium difficile infection; cipro, ciprofloxacin; ED, emergency department; FQ, fluoroquinolone; gati, gatifloxacin; IQR, interquartile range; levo, levofloxacin; moxi, moxifloxacin; NA, not applicable, NR, not reported; SCAR, severe cutaneous adverse reaction

### Health service use

Although the search covered all AEs related to FQs, the AEs depicted in the included studies can mostly be defined as serious, since hospitalization was the most frequently reported AE-related health service use (17 studies [20]-[30][25]-[35][36][37]). Hospitalization was required in all CDAD -cases and serious cutaneous AEs. McFarland et al. provided the most detailed report of health service use relating to CDAD. In the study 30 percent of CDAD -patients were admitted to an ICU, two percent required surgical intervention and 21 percent were readmitted to a health care facility [22]. The specific number of hospitalized patients was not detailed in the included studies. Fatalities were reported in several studies ([20][24][28][32][35]-[37]). However, none of the fatalities were directly associated with FQs. FQ-related cutaneous AEs were highlighted specifically in studies of Asian origin ([30][33][37]).

In addition, emergency department (ED) visits were reported in four studies ([21][26][38][29]). Length of hospital stay was reported in 10 studies ([20][22][24][26] [30][32][34][35][36][37]) and varied between <5 and 45 days. Long hospital stays were particularly associated with CDAD.

### Costs

AE-related costs were evaluated and reported in only five studies ([23][25][32][35][29]) and the disparity between estimations was significant. The cost of an AE-related episode varied in this systematic review between 140 and 18,252 € and there was also considerable variation among AE episodes within some individual studies. Llop, for example, evaluated the cost of an average FQ-related AE episode to be 4,528±18,252 € [25]. In this systematic review, the highest reported health care costs were associated with CDAD, and costs associated with other AEs were not specified. In four studies, costs were evaluated from the perspective of the hospital ([23][32][35][29]). Mjörndal et.al. [32] and Perrone et.al. [29] specifically stated that costs consist of direct hospital costs. Llop et.al.[25] did not specify cost details beyond costs associated with AEs and retreatment. None of the included studies reported travel or time costs, indirect costs or specified the payer.

### Differences in adverse events according to various fluoroquinolones

Levofloxacin[20]-[27][30][25][28][29][36] and ciprofloxacin [20][22]-[27][38][32][33][35][36][37] were the most frequently utilized interventions (Table 3), with both being included in 12 studies. In these studies, levofloxacin was associated with various AEs, including dysglycemia, CDAD, hepatotoxicity, diarrhea, altered mental status, rash and thrush. AEs associated with ciprofloxacin included dysglycemia, CDAD, hepatotoxicity, hepatitis, Stevens-Johnson Syndrome (SJS), acute generalized exanthematous pustulosis (AGEP), increased prothrombin complex, seizures, diarrhea, rash and fever. Moxifloxacin was included in four studies[20][21][24][26] and associated with dysglycemia, CDAD and hepatotoxicity. Norfloxacin[31] was present in one study and linked to hepatitis. Ofloxacin use was reported in five studies [30][38][33]-[35] and linked to an epileptic seizure, urticarial lesion, fixed drug effect, exfoliative dermatitis, angioedema and photodermatitis (PD).

**Table 3:** Health service use and costs associated with FQ-related AEs.

AE type	Study	Intervention(s) relevant to study	Reported FQ AE	AE occurence	Fatalities in study associated with any AE	AE-related health service use	Length of hospital stay	AE costs^a^
**Dysglycemia**	Aspinall et al. (2009) [27]	Levo, cipro	N = 212 Hypoglycemia: levo n = 86, cipro n = 19; hyperglycemia: levo n = 84, cipro n = 23	Incidence per 1,000 patients: Hypoglycemia: levo 0.19 (95% CI 0.15–0.23), cipro 0.10 (0.06–0.15), hyperglycemia: levo 0.18 (0.15–0.23), cipro 0.12 (0.08–0.18)	None reported	Hospitalization	NR	NR
	Chou et al (2013) [26]	Cipro, levo, moxi	N = 375 Hypoglycemia: cipro n = 99, levo n = 109, moxi n = 42; hyperglycemia: cipro n = 50, levo n = 46, moxi n = 29	Incidence per 1,000 patients: Hypoglycemia: cipro 7.88, levo 9.26, moxi 9.95, hyperglycemia: cipro 3.98, levo 3.91, moxi 6.87	None reported	ED visit or hospitalization	Median, days, hypoglycemia: cipro 15, levo 9, moxi 14; hyperglycemia: cipro 12, levo 10, moxi 13	NR
**CDAD**	Dhalla et al. (2006)[20]	Levo, cipro, moxi	N = 88 Levo n = 28, cipro n = 44, moxi n = 16	OR (95% CI): Levo (reference) 1.00, cipro 0.85 (0.52–1.41), moxi 1.18 (0.61–2.27)	N = 16 (16,7%)	Hospitalization	Median 12 days (IQR 6–23)	NR
	Mah et al. (2011) [28]	Levo	N = 66	2.5%	N = 10/202 (5%) died or had a colectomy	Hospitalization	NR	NR
	McFarland et al. (2007) [22]	Levo, cipro	N = 41 Levo n = 33, cipro n = 8	Unclear	N = 54 (15%)	Hospitalization: 30% required ICU and 21% readmission to a health care facility <1 year after hospital discharge, 2% of patients required gastrointestinal surgery	Total mean days ± SD 45.2 (6.3)	NR
	Muto et al. (2005) [23]	Levo, cipro	N = 135 Levo n = 120, cipro n = 15	Levo OR (95% CI) 2.0 (1.2–3.3)	N = 18	Hospitalization	NR	3,571 €/episode, health care costs due to CDAD outbreak 2000–2001 903,407 €
**Liver injury or hepatitis**	Kaye et al. (2014) [21]	Levo, moxi	N = 175 Liver injury associated with levo within 30 days of exposure n = 58, moxi n = 30, liver injury associated with levo within 90 days of exposure n = 57, moxi n = 25, liver failure levo n = 5	Liver injury incidence per 100,000 person-years associated with levo within 30 days of exposure 134.3, moxi 116.4, incidence associated with levo within 90 days of exposure 70.9, moxi 52.6	N = 32 (5.3%)	Hospitalization, ED visit	NR	NR
	Martí et al. (2005) [31]	Nor	Hepatitis n = 2	Unclear	None reported	Hospitalization	NR	NR
	Paterson et al. (2012)[24]	Cipro, levo, moxi	N = 121 Hepatotoxicity associated with cipro n = 67, levo n = 28, moxi n = 26	Incidence per 100, 000 exposures cipro: 6.37, levo: 8.62, moxi: 7.89	N = 88 (61.1%)	Hospitalization	Median 8 (IQR 4–16) days	NR
**Seizure**	Olivier et al. (2002) [34]	Oflo	Epileptic seizure n = 1	Unclear	None reported	Hospitalization	10 days	NR
**Cutaneous AEs**	Jamunarani and Priya (2014) [30]	Levo, oflo	Maculopapular eruption (levo), urticarial lesion (oflo), fixed drug effect (oflo), exfoliative dermitis (oflo), angioedema (oflo), n = 8	Unclear	None reported	Hospitalization	< 5 days 21.2%, 5–20 days 63.6%, > 20 days 15.2%	NR
	Noel et al. (2004) [33]	Cipro, Oflo	N = 2 SJS (cipro n = 1), PD (oflo n = 1)	Unclear	None reported	Hospitalization	NR	NR
	Su and Aw (2014)[37]	Cipro	SJS, AGEP n = 2	Unclear	N = 1	Hospitalization	SJS: 34 days, AGEP: 16 days	NR
**Several reported AEs**	Llop et al. (2017) *[25]	Levo (68%), other FQ (32%)	N = 26,893 Clostridium difficile infection and enterocolitis n = 122, peripheral neuropathy n = 375, tendinitis n = 1,326, digestive effects n = 5,667, CNS effects n = 14,951, skin reactions n = 2,516, hepatotoxicity n = 543, hematologic toxicity n = 6,540	16.2%	None reported	Hospitalization	NR	Unadjusted costs of AE 4,528 € ± 18,252 €
	Mjörndal et al. (2002) [32]	Cipro	N = 2 Increased prothrombin complex n = 1, seizures n = 1	Unclear	N = 2	Hospitalization	6 (0–30) days	Average cost of treating one person with ADR 2,700 €
	Patel et al. (2007)[35]	Cipro, Oflo	Complex partial seizures, peripheral neuropathy, dystonia, hypersensitivity reaction, tendinitis, dysgeusia; n = unclear	Unclear	N = 17 (0.83%)	Hospitalization	Median 5 days (95% CI 5.37–7.11)	Average cost per patient hospitalized 140 €
	Sánchez Muñoz-Torrero et al. (2010) [36]	Levo, cipro	N = 32 Diarrhea (levo n = 17, cipro n = 4), pseudomembranous colitis (levo n = 2), altered mental status (levo n = 1), rash (levo n = 1, cipro n = 1), thrush (levo n = 4), hepatitis (cipro n = 1), fever (cipro n = 1)	Unclear	N = 2 (1,6%)	Hospitalization	Median 18 ±17 days	NR
**Non-specified AEs**	Jayarama et al. (2012) [38]	Cipro, Oflo	N = 3 Cipro n = 2, oflo n = 1	Unclear	None reported	ED visit	NA	NR
	Perrone et al. (2014) [29]	Levo	N = 172	Unclear	1,5%	ED visit	NA	Mean 592 € ± 2,175 € / patient

* Out-patient analysis; AE, adverse event; AGEP, acute generalized exanthematous pustulosis; CDAD, clostridium difficile-associated diarrhea; cipro, ciprofloxacin; ED, emergency department; FQ, fluoroquinolone; levo, levofloxacin; moxi, moxifloxacin; nor, norfloxacin; NA, not applicable; NR, not reported; oflo, ofloxacin; OR, odds ratio; SD, standard deviation; SJS, Stevens-Johnson syndrome

^a^All costs converted into 2016 euro

In the included studies, norfloxacin and ofloxacin were associated with the least reports of health service use and costs. Conversely, levofloxacin and ciprofloxacin, the most frequently considered FQs, appeared to be connected to the most AEs, health service use and costs. Health service use and health service costs associated with FQ-related AEs are depicted in Table 3.

### The quality of the included studies

**Fig 2:**
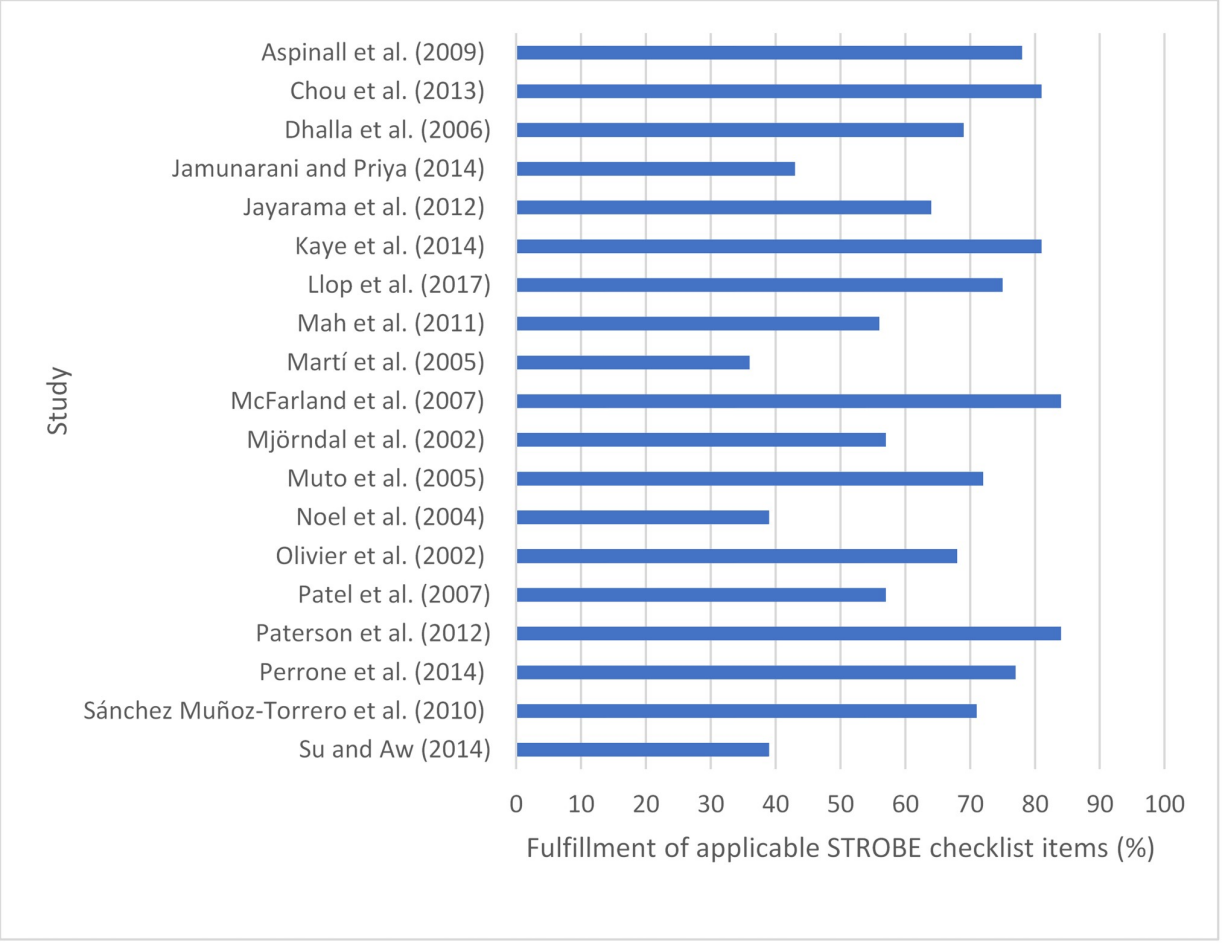
Quality assessment of the included studies. The included studies were assessed according to STROBE checklist and awarded scores, which are presented in percentages.

The results of the quality assessment are illustrated in Fig 2. The included studies scored an average 19.74 and median 20 (range 10 and 27) points out of 34 total points. The weighted average rating was 65% (range 36–84%). Although the scores are relatively high, some inadequacies were apparent in reporting. Only six studies described efforts to address potential sources of bias ([20]-[22][24][26][27]). Two studies provided an explanation for the population sample size ([22][34]).

Seven studies failed to report the funding of the study ([30][38][31][33][35][36][37]). The case-controlled observational studies all reported the source of research funding but otherwise there was no difference in the results of the quality assessment regarding study design. The fulfillment of the STROBE checklist items is portrayed in S1 Table.

## Discussion

The aim of this systematic review was to identify health service use and costs associated with FQ-related AEs. To date, research concentrating on costs associated with drug-related AEs remains scarce. As far as we know, the economic impacts of any FQ-related AEs have previously not been examined in a systematic review. Due to the substantial gap in published literature, we were unable to examine many serious and costly FQ-related AEs, such as neuropsychiatric AEs, QT interval prolongation, aortic aneurysm and tendinopathy in this review. There was considerable heterogeneity among the included studies. The most variation was associated with population sample sizes (n = 33–1,277,248) and study duration (4 weeks—22 years) as well as AEs considered. Although randomized controlled trials (RCTs) were not excluded from the literature search, all the included studies were observational. Observational studies may pick up on AEs not observed in RCTs, which might be due to several factors. RCTs frequently exclude patients who are most vulnerable to AEs, such as the elderly and patients with comorbidities. In addition, sample sizes are in many cases smaller and follow-up periods often shorter in RCTs than in observational studies. Of the 19 studies included in the review, five were case-controlled, in order to explicitly observe risk rates of AEs associated with FQs. Even then, the number of FQ-related AEs assessed in the included studies in proportion to the population size was small, which could mean that all FQ-related AEs were not assessed. In 13 studies[20]-[24][26]-[30][28][31][33][34][37], only specific AEs were examined and many AEs may not have been reported or even recognized. Of the five FQs in this study, levofloxacin was associated with the most reported AEs, health service use, length of hospital stay and costs. Ciprofloxacin was associated with similar AEs, health service use, length of stay and costs as levofloxacin, but with smaller volume. Norfloxacin, on the other hand, was only linked to two cases of hepatitis. These data do not allow comparisons across FQs and drawing of definite conclusions relating to health service use and costs associated with levofloxacin, ciprofloxacin, moxifloxacin, norfloxacin and ofloxacin. Levofloxacin and ciprofloxacin were considered in 12 studies, including extremely large studies, and norfloxacin in only one. Therefore, the number of AEs associated with specific FQs reported in the studies is related to the utilization of the FQ and not necessarily to the toxicity. At present ciprofloxacin followed by levofloxacin are the most consumed FQs globally[39][40]. Previous research has shown that the safety profiles of the FQs included in this systematic review are similar to each other[1].

In this systematic review, hospitalizations and ED visits were the main health service use outcomes associated with AEs. Outpatient visits to primary care facilities were not reported in the included studies, although it is likely that most AEs are diagnosed and treated in primary care, if recognized as FQ-related at all. According to prior research by Magdelijns et.al., hospitalizations, specifically long stays in hospital, are the leading cost drivers in health service use. Hospitalizations were estimated to cause approximately 77% of direct health care costs associated with AEs in the Netherlands[41].

Reported FQ-related AE-costs varied between 140 and 18,252€ per AE episode. CDAD was associated with the largest amount of health service use, longest stays in hospital and, thus, the highest reported costs of AEs considered. Mean CDAD-related length of stays were up to 45 days. Since the emergence of the epidemic *Clostridium difficile* ribotype 027 clone, CDAD has become more prevalent, severe and more difficult to treat, due to resistance to many antimicrobial agents[42]. The included studies took only into account the direct treatment costs, which does not represent the total costs of a FQ-related AE episode. Evaluating all AE-triggered costs, regardless of who they fall on, would reflect a more accurate assessment. However, as described in Table 2, the aims of the included studies did not involve examining health service use or costs. Therefore, both health service use and costs were addressed in a cursory manner and were likely underestimated. In the five studies that did report costs ([23][25][32][35][29]), they proved difficult to compare. Costs relating to healthcare systems, diagnostic methods and treatment protocols differ significantly depending on the origin of the study and the AEs considered. In addition, the severity of the reported FQ-related AEs may have fluctuated and resulted in diverse health service use and costs. AE-related costs, when reported, lack adequate transferability. Conversely, health service use and length of hospital stay are outcomes that can be more effectively compared and transferred, regardless of the origin of the study. Even here, temporal, geographical and payer differences may lead to disparities in these metrics for similar AEs.

Limitations of this systematic review include confining the literature search to full English language texts. However, the risk of lost key findings is minor due to the paucity of non-English texts excluded from the review. In addition, we excluded studies with pediatric patients, though inclusion could have led to added information about health service use and costs. The use of FQs in children continues to be limited or restricted. Although studies have described the majority of FQ-related AEs in pediatric patients as temporary and reversible[43], real-world safety data continue to be scarce. We acknowledge that the use of STROBE checklist for observational studies is not recommended for assessing the methodological quality of studies. There is a distinct deficiency of reliable, comprehensive and validated tools for the quality assessment of observational studies. We did not exclude any studies due to poor quality and therefore using STROBE did not introduce bias into this systematic review. Additionally, there is a lack of guidelines and definitions regarding data quality, which is not addressed in quality assessments. This could potentially cause bias. The shortage of existing research relating to health service use and costs associated with FQ-related AEs and the incomplete nature of AEs considered in those that do report these, account for the largest limitation of this systematic review. Funding, in addition to the undetection and underreporting of AEs are issues that can restrict and direct studies. Present means and resources available to allow independent AE-research are poor.

## Conclusions

Because of the wide clinical use of FQs, in particular serious FQ-related AEs can have substantial economic implications, in addition to imposing potentially long-lasting health complications for patients. Better-quality reporting and additional published data on health service use and costs associated with AEs are both necessary and overdue.

## Supporting information

S1 Table Fulfillment of Items of quality assessment. (PDF)

S1 File Search strategy. (PDF)

S2 File List of excluded articles. (PDF)

S1 Checklist PRISMA checklist. (PDF)
