# Supplementary material for: Fluoroquinolone-related adverse events resulting in health service use and costs: A systematic review
Source: PLoS One. 2019 Apr 26;14(4):e0216029. doi: 10.1371/journal.pone.0216029 (PMC6485715; doi:10.1371/journal.pone.0216029)
Supplement: S1 File — (PDF) [file pone.0216029.s002.pdf]

S2 File. Search strategy.

Search strategy for Medline (Ovid)
Date of the search April 6, 2017

1. exp Fluoroquinolones/ or fluoroquinolone*.mp.
2. exp Ciprofloxacin/ or ciprofloxacin*.mp.
3. exp Norfloxacin/ or norfloxacin*.mp.
4. exp Levofloxacin/ or levofloxacin*.mp.
5. exp Ofloxacin/ or ofloxacin*.mp.
6. moxifloxacin*.mp.
7. 1 or 2 or 3 or 4 or 5 or 6
8. exp Fluoroquinolones/ae, po, to [Adverse Effects, Poisoning, Toxicity]
9. exp Ciprofloxacin/ae, po, to [Adverse Effects, Poisoning, Toxicity]
10. exp Norfloxacin/ae, to [Adverse Effects, Toxicity]
11. exp Levofloxacin/ae, to [Adverse Effects, Toxicity]
12. exp Ofloxacin/ae, po, to [Adverse Effects, Poisoning, Toxicity]
13. exp "Drug-Related Side Effects and Adverse Reactions"/
14. exp Adverse Drug Reaction Reporting Systems/
15. exp Pharmacovigilance/
16. (adverse adj3 (effect* or event* or reaction*)).mp. [mp=title, abstract, original title, name of substance word, subject heading word, keyword heading word, protocol supplementary concept word, rare disease supplementary concept word, unique identifier, synonyms]
17. side effect*.mp.
18. toxicit*.mp.
19. exp Tendon Injuries/ or tendon injur*.mp.
20. (tendin* or tendon*).mp. [mp=title, abstract, original title, name of substance word, subject heading word, keyword heading word, protocol supplementary concept word, rare disease supplementary concept word, unique identifier, synonyms]
21. exp Tendinopathy/
22. neuropath*.mp.
23. clostridium difficile.mp. or exp Clostridium difficile/
24. exp "Chemical and Drug Induced Liver Injury"/
25. exp Long QT Syndrome/
26. exp Psychotic Disorders/ or psychosis*.mp.
27. exp Seizures/ or seizure*.mp.
28. exp Retinal Detachment/
29. exp Hypersensitivity/ or hypersensitivit*.mp.
30. 8 or 9 or 10 or 11 or 12 or 13 or 14 or 15 or 16 or 17 or 18 or 19 or 20 or 21 or 22 or 23 or 24 or 25 or 26 or 27 or 28 or 29
31. exp "Length of Stay"/
32. exp Hospitalization/
33. (hospitalized adj3 (day or charge*)).mp. [mp=title, abstract, original title, name of substance word, subject heading word, keyword heading word, protocol supplementary concept word, rare disease supplementary concept word, unique identifier, synonyms]
34. (hospital adj3 (visit* or stay)).mp. [mp=title, abstract, original title, name of substance word, subject heading word, keyword heading word, protocol supplementary concept word, rare disease supplementary concept word, unique identifier, synonyms]
35. healthcare.mp.
36. (health adj3 (care or service)).mp. [mp=title, abstract, original title, name of substance word, subject heading word, keyword heading word, protocol supplementary concept word, rare disease supplementary concept word, unique identifier, synonyms]
37. (hospital adj3 admission*).mp. [mp=title, abstract, original title, name of substance word, subject heading word, keyword heading word, protocol supplementary concept word, rare disease supplementary concept word, unique identifier, synonyms]
38. (outpatient adj3 (visit* or service*)).mp. [mp=title, abstract, original title, name of substance word, subject heading word, keyword heading word, protocol supplementary concept word, rare disease supplementary concept word, unique identifier, synonyms]

39. (inpatient adj3 stay).mp. [mp=title, abstract, original title, name of substance word, subject heading word, keyword heading word, protocol supplementary concept word, rare disease supplementary concept word, unique identifier, synonyms]
40. (emergency adj3 (room or department)).mp. [mp=title, abstract, original title, name of substance word, subject heading word, keyword heading word, protocol supplementary concept word, rare disease supplementary concept word, unique identifier, synonyms]
41. (ED or ER).mp. [mp=title, abstract, original title, name of substance word, subject heading word, keyword heading word, protocol supplementary concept word, rare disease supplementary concept word, unique identifier, synonyms]
42. exp Economics, Pharmaceutical/ or exp Economics, Medical/ or exp Economics, Hospital/ or exp Economics, Nursing/
43. economic*.mp.
44. pharmacoeconomic*.mp.
45. cost*.mp. or exp "Costs and Cost Analysis"/ or exp Cost-Benefit Analysis/ or exp "Cost of Illness"/
46. expense*.mp.
47. charge*.mp.
48. (economic* adj3 burden*).mp. [mp=title, abstract, original title, name of substance word, subject heading word, keyword heading word, protocol supplementary concept word, rare disease supplementary concept word, unique identifier, synonyms]
49. expenditure*.mp.
50. admission*.mp.
51. 31 or 32 or 33 or 34 or 35 or 36 or 37 or 38 or 39 or 40 or 41 or 42 or 43 or 44 or 45 or 46 or 47 or 48 or 49 or 50
52. 7 and 30 and 51
53. remove duplicates from 52
