# Supplementary material for: Fluoroquinolone-related adverse events resulting in health service use and costs: A systematic review
Source: PLoS One. 2019 Apr 26;14(4):e0216029. doi: 10.1371/journal.pone.0216029 (PMC6485715; doi:10.1371/journal.pone.0216029)
Supplement: S2 File — (PDF) [file pone.0216029.s003.pdf]

S3 File. Studies excluded after full-text assessment. Duplicate references (n=8) are excluded from the list.

1. (CDC) C for DC and P. Surveillance for community-associated Clostridium difficile--Connecticut, 2006. MMWR Morb Mortal Wkly Rep. 2008;57(13):340-343.

2. Actis GC, Pellicano R, Fadda M, Rosina F. Antibiotics and non-steroidal anti-inflammatory drugs in outpatient practice: Indications and unwanted effects in a gastroenterological setting. Curr Drug Saf. 2014;9(2):133-137.

3. Aldeyab MA, Noble SC, Cuthbert M, Maxwell S, Dear J, Boyter A. Assessment of the impact of the Scottish public health campaign on patient reporting of adverse drug reactions. Drugs Ther Perspect. 2016;32(5):209-218.

4. Al-Saadi S, Michael A. Levofloxacin-induced Achilles tendinitis and tendon rupture. Eur Geriatr Med. 2012;3(6):380-381.

5. Alsbou M. Incidence of adverse drug reactions in alkarak hospital: A pilot study. Jordan Med J. 2010;44(4):442-446.

6. Alsbou M, Alzubiedi S, Alzobi H, et al. Adverse drug reactions experience in a teaching hospital in Jordan. Int J Clin Pharm. 2015;37(6):1188-1193.

7. Alshammari TM, Larrat EP, Morrill HJ, Caffrey AR, Quilliam BJ, LaPlante KL. Risk of hepatotoxicity associated with fluoroquinolones: a national case-control safety study. Am J Health Syst Pharm. 2014;71(1):37-43.

8. Artukovic M, Kustelega J, Lugovic-Mihic L. DRESS syndrome with mild manifestations as a diagnostic and therapeutic problem: case report. Acta Clin Croat. 2010;49(4):479-484.

9. Avery LM, Zempel M, Weiss E. Case of antibiotic-associated diarrhea caused by Staphylococcus aureus enterocolitis. Am J Health Syst Pharm. 2015;72(11):943-951.

10. Babar SM. SIADH associated with ciprofloxacin. Ann Pharmacother. 2013;47(10):1359-1363.

11. Balfour JA, Faulds D. Oral Ciprofloxacin: A Pharmacoeconomic Evaluation of its Use in the Treatment of Serious Infections. Pharmacoeconomics. 1993;3(5):398-421.

12. Ball P. Safety of the new fluoroquinolones compared with ciprofloxacin. J Chemother. 2000;12(SUPPL. 1):8-11.

13. Ball P. Efficacy and safety of Levofloxacin in the context of other contemporary fluoroquinolones: A review. Curr Ther Res Exp. 2003;64(9):646-661.

14. Ball P. New antibiotics for community-acquired lower respiratory tract infections: improved activity at a cost? Int J Antimicrob Agents. 2000;16(3):263-272.

15. Ball P. Adverse drug reactions: implications for the development of fluoroquinolones. J Antimicrob Chemother. 2003;51 Suppl 1:21-27.

16. Ballow CH. Cost considerations in oral antibiotic therapy. Adv Ther. 1995;12(4):199-206.

17. Bansal N, Manocha D, Madhira B. Life-Threatening Metabolic Coma Caused by Levofloxacin. Am J Ther. 2015;22(2):e48-e51.

18. Barriere SL. Economic impact of oral ciprofloxacin. A pharmacist's perspective. Am J Med. 1987;82(4A):387-390.

19. Barvaliya M, Sanmukhani J, Patel T, Paliwal N, Shah H, Tripathi C. Drug-induced Stevens-Johnson syndrome (SJS), toxic epidermal necrolysis (TEN), and SJS-TEN overlap: A multicentric retrospective study. J Postgrad Med. 2011;57(2):115-119.

20. Baxter R, Ray GT, Fireman BH. Case-control study of antibiotic use and subsequent Clostridium difficile-associated diarrhea in hospitalized patients. Infect Control Hosp Epidemiol. 2008;29(1):44-50.

21. Bellon A, Perez-Garcia G, Coverdale JH, Chacko RC. Seizures associated with levofloxacin: Case presentation and literature review. Eur J Clin Pharmacol. 2009;65(10):959-962.

22. Beringer PM, Wong-Beringer A, Rho JP. Economic aspects of antibacterial adverse effects. Pharmacoeconomics. 1998;13(1 Pt 1):35-49.

23. Bhattacharyya S, Darby RR, Raibagkar P, Castro LNG, Berkowitz AL. Antibiotic-associated encephalopathy. Neurology. 2016;86(10):963-971.

24. Biller P, Shank B, Lind L, et al. Moxifloxacin therapy as a risk factor for Clostridium difficile-associated disease during an outbreak: Attempts to control a new epidemic strain. Infect Control Hosp Epidemiol. 2007;28(2):198-201.

25. Bird ST, Etminan M, Brophy JM, Hartzema AG, Delaney JAC. Risk of acute kidney injury associated with the use of fluoroquinolones. CMAJ. 2013;185(10):E475-82.

26. Boesler B, Ott M, Menges M. Segmental haemorrhagic colitis under ciprofloxacin therapy. Z Gastroenterol. 2009;47(5):429-431.

27. Borgmann S, Jakobiak T, Gruber H, et al. Association of ciprofloxacin prescriptions to outpatients to Clostridium difficile infections. EUROSURVEILLANCE. 2010;15(5):13-15.

28. Botelho-Nevers E, Rovery C, Richet H, Raoult D. Analysis of risk factors for malignant Mediterranean spotted fever indicates that fluoroquinolone treatment has a deleterious effect. J Antimicrob Chemother. 2011;66(8):1821-1830.

29. Brown KA, Khanafer N, Daneman N, Fisman DN. Meta-Analysis of Antibiotics and the Risk of Community-Associated Clostridium difficile Infection. Antimicrob Agents Chemother. 2013;57(5):2326-2332.

30. Bruns AHW, Oosterheert JJ, Kuijper EJ, et al. Impact of different empirical antibiotic treatment regimens for community-acquired pneumonia on the emergence of Clostridium difficile. J Antimicrob Chemother. 2010;65(11):2464-2471.

31. Caldwell JW, Singh S, Johnson RH. Clinical and economic evaluation of subsequent infection following intravenous ciprofloxacin or imipenem therapy in hospitalized patients with severe pneumonia. J Antimicrob Chemother. 1999;43 Suppl A:129-134.

32. Capuano A, Irpino A, Gallo M, et al. Regional surveillance of emergency-department visits for outpatient adverse drug events. Eur J Clin Pharmacol. 2009;65(7):721-728.

33. Capuano A, Motola G, Russo F, et al. Adverse drug events in two emergency departments in Naples, Italy: An observational study. Pharmacol Res. 2004;50(6):631-636.

34. Carrascosa MF, Lucena MI, Andrade RJ, et al. Fatal acute hepatitis after sequential treatment with levofloxacin, doxycycline, and naproxen in a patient presenting with acute Mycoplasma pneumoniae infection. Clin Ther. 2009;31(5):1014-1019.

35. Cereza G, Agustí A, Pedrós C, et al. Effect of an intervention on the features of adverse drug reactions spontaneously reported in a hospital. Eur J Clin Pharmacol. 2010;66(9):937-945.

36. Chan EW, Liu KQL, Chui CSL, Sing C-W, Wong LYL, Wong ICK. Adverse drug reactions - Examples of detection of rare events using databases. Br J Clin Pharmacol. 2015;80(4):855-861.

37. Chang KC, Leung CC, Yew WW, et al. Analyses of fluoroquinolones and Clostridium difficile-associated diarrhoea in tuberculosis patients. Int J Tuberc Lung Dis. 2009;13(3):341-346.

38. Changela U, Cannon JP, Aneziokoro C, Shah PS, Thottapurathu L, Lentino J. Risk factors and mortality associated with Clostridium difficile-associated diarrhoea at a VA hospital. Int J Antimicrob Agents. 2004;24(6):562-566.

39. Chou H-W, Wang J-L, Chang C-H, Lai C-L, Lai M-S, Chan KA. Risks of cardiac arrhythmia and mortality among patients using new-generation macrolides, fluoroquinolones, and beta-lactam/beta-lactamase inhibitors: a Taiwanese nationwide study. Clin Infect Dis. 2015;60(4):566-577.

40. Christie MJ, Wong K, Ting RH, Tam PY, Sikaneta TG. Generalized seizure and toxic epidermal necrolysis following levofloxacin exposure. Ann Pharmacother. 2005;39(5):953-955.

41. Chung P, Currie B, Guo Y, Talansky M, Brown S, Ostrowsky B. Investigation to identify a resource-efficient case-control methodology for determining antibiotics associated with Clostridium difficile infection. Am J Infect Control. 2014;42(10 Suppl):S264-8.

42. Çoban Ş, Ceydilek B, Ekiz F, Erden E, Soykan I. Levofloxacin-induced acute fulminant hepatic failure in a patient with chronic hepatitis B infection. Ann Pharmacother. 2005;39(10):1737-1740.

43. Cohen JS. Peripheral neuropathy associated with fluoroquinolones. Ann Pharmacother. 2001;35(12):1540-1547.

44. Courjon J, Pulcini C, Cua E, et al. Antibiotics-related adverse events in the infectious diseases department of a French teaching hospital: A prospective study. Eur J Clin Microbiol Infect Dis. 2013;32(12):1611-1616.

45. Cox ZL, McCoy AB, Matheny ME, et al. Adverse drug events during AKI and its recovery. Clin J Am Soc Nephrol. 2013;8(7):1070-1078.

46. Cupurdija V, Lazic Z, Jankovic S, et al. Adverse events induced by anti-infectives in hospitalized patients. Serbian J Exp Clin Res. 2011;12(3):97-101.

47. Darwish T. Ciprofloxacin-induced seizures in a healthy patient. N Z Med J. 2008;121(1277):104-105. 48. Davies BI, Maesen FP V, Teengs JP, Baur C. The quinolones in chronic bronchitis. Pharm Weekbl Sci Ed. 1986;8(1):53-59. doi:10.1007/BF01975481.

49. Davies EC, Green CF, Mottram DR, Pirmohamed M. Adverse drug reactions in hospital in-patients: A pilot study. J Clin Pharm Ther. 2006;31(4):335-341.

50. Davies EC, Green CF, Taylor S, Williamson PR, Mottram DR, Pirmohamed M. Adverse drug reactions in hospital in-patients: A prospective analysis of 3695 patient-episodes. PLoS One. 2009;4(2).

51. de Bazignan AD, Thiessard F, Miremont-Salame G, Conri C, Haramburu F, Pharmacovigilance RCR. Fluoroquinolone psychiatric adverse effects: review of cases from the french pharmacovigilance database. Rev Med INTERNE. 2006;27(6):448-452.

52. Deshpande A, Pant C, Jain A, Fraser TG, Rolston DDK. Do fluoroquinolones predispose patients to Clostridium difficile associated disease? A review of the evidence. Curr Med Res Opin. 2008;24(2):329-333.

53. Devi K, George S, Narayanan B. A study of severe cutaneous adverse reactions to drugs with special reference to treatment outcome. Indian J Dermatol Venereol Leprol. 2016;82(2):239.

54. Dhasmana DC, Seth V, Mishra KC. Voluntary adverse drug reaction reporting in a tertiary care teaching hospital. Indian J Pharmacol. 2002;34(3):204-205.

55. Dial S, Kezouh A, Dascal A, Barkun A, Suissa S. Patterns of antibiotic use and risk of hospital admission because of Clostridium difficile infection. CMAJ. 2008;179(8):767-772.

56. Diphoorn J, Cazzaniga S, Gamba C, et al. Incidence, causative factors and mortality rates of Stevens-Johnson syndrome (SJS) and toxic epidermal necrolysis (TEN) in northern Italy: Data from the REACT registry. Pharmacoepidemiol Drug Saf. 2016;25(2):196-203.

57. Dissemination C for R and. Advantages of moxifloxacin and levofloxacin-based triple therapy for second-line treatments of persistent Helicobacter pylori infection: a meta analysis (Structured abstract). Database Abstr Rev Eff. 2015;(2).

58. Dissemination C for R and. Meta-analysis of antibiotics and the risk of community-associated Clostridium difficile infection (Structured abstract). Database Abstr Rev Eff. 2015;(2).

59. Durey A, Baek YS, Park JS, et al. Levofloxacin-induced achilles tendinitis in a young adult in the absence of predisposing conditions. Yonsei Med J. 2010;51(3):454-456.

60. Elliott TR, Symes T, Kannourakis G, Angus P. Resolution of norfloxacin-induced acute Liver failure after N-acetylcysteine therapy: Further support for the use of NAC in drug-induced ALF? BMJ Case Rep. 2016;2016.

61. Famularo G, Pizzicannella M, Gasbarrone L. Levofloxacin and seizures: What risk for elderly adults? J Am Geriatr Soc. 2014;62(10):2018-2019.

62. Farhat S, Banday M, Hassan I. Antecedent drug exposure aetiology and management protocols in Steven-Johnson syndrome and toxic epidermal necrolysis, A hospital based prospective study. J Clin Diagnostic Res. 2016;10(1):FC01-FC04.

63. Fennig S, Mauas L. Ofloxacin-induced delirium. J Clin Psychiatry. 1992;53(4):137-138.

64. Ferner RE, Butt TF. Adverse drug reactions. Med (United Kingdom). 2012;40(7):366-370.

65. Fife D, Zhu V, Voss E, Levy-Clarke G, Ryan P. Exposure to oral fluoroquinolones and the risk of retinal detachment: retrospective analyses of two large healthcare databases. Drug Saf. 2014;37(3):171-182.

66. Figueira-Coelho J, Pereira O, Picado B, Mendonca P, Neves-Costa J, Neta J. Acute hepatitis associated with the use of levofloxacin. Clin Ther. 2010;32(10):1733-1737.

67. Foti C, Romita P, Zanframundo G, et al. Ciprofloxacin induced acute generallised exanthematous pustulosis. Indian J Pharmacol. 2017;49(1):119-120.

68. Franchi C, Ardoino I, Rossio R, et al. Prevalence and Risk Factors Associated with Use of QT-Prolonging Drugs in Hospitalized Older People. Drugs and Aging. 2016;33(1):53-61.

69. Gabutti L, Stoller R, Marti HP. [Fluoroquinolones as etiology of tendinopathy]. Fluoroquinolone als Ursache von Tendinopathien. 1998;55(9):558-561.

70. Gallagher JC, Du JK, Rose C. Severe Pseudomembranous Colitis After Moxifloxacin Use: A Case Series. Ann Pharmacother. 2009;43(1):123-128.

71. Gallelli L, Ferreri G, Colosimo M, et al. Adverse drug reactions to antibiotics observed in two pulmonology divisions of Catanzaro, Italy: A six-year retrospective study. Pharmacol Res. 2002;46(5):395-400.

72. Ganske CM, Horning KK. Levofloxacin-induced tendinopathy of the hip. Ann Pharmacother. 2012;46(5):e13.

73. Garber SM, Pound MW, Miller SM. Hypoglycemia associated with the use of levofloxacin. Am J Health Syst Pharm. 2009;66(11):1014-1019.

74. Gholami K, Parsa S, Shalviri G, Sharifzadeh M, Assasi N. Anti-infectives-induced adverse drug reactions in hospitalized patients. Pharmacoepidemiol Drug Saf. 2005;14(7):501-506.

75. Ghoshal A, Damani A, Salins N, Deodhar J, Muckaden MA. Management of Levofioxacin Induced Anaphylaxis and Acute Delirium in a Palliative Care Setting. Indian J Palliat Care. 2015;21(1):76-78.

76. Gleckman RA, Czachor JS. Antibiotic side effects. Semin Respir Crit Care Med. 2000;21(1):53-60.

77. Goff DA. Determining the cost of antimicrobial therapy: A focus on fluoroquinolones. FORMULARY. 1998;33(7):648+.

78. Gomes ER, Demoly P. Epidemiology of hypersensitivity drug reactions. Curr Opin Allergy Clin Immunol. 2005;5(4):309-316.

79. Gómez ES, Martínez MTG, Leñero VD, Ledesma MB, Saiz SG, Martín CB. Active pharmacovigilance in a hospital pharmacy department. EJHP Pract. 2009;15(4):33-36.

80. Grimm O, Alm B. A case of ciprofloxacin-induced acute polymorphic psychosis with a distinct deficit in executive functions [1]. Psychosomatics. 2007;48(3):269.

81. Grossman R, Mukherjee J, Vaughan D, et al. A 1-year community-based health economic study of ciprofloxacin vs usual antibiotic treatment in acute exacerbations of chronic bronchitis: the Canadian Ciprofloxacin Health Economic Study Group. Chest. 1998;113(1):131-141.

82. Guharoy SR. Serum sickness secondary to ciprofloxacin use. Vet Hum Toxicol. 1994;36(6):540-541.

83. Gulen M, Ay MO, Avci A, Acikalin A, Icme F. Levofloxacin-induced hepatotoxicity and death. Am J Ther. 2015;22(3):e93-6.

84. Gupta A, Guron N, Harris M, Bell R. Levofloxacin-induced rhabdomyolysis in a hemodialysis patient. Hemodial Int. 2012;16(1):101-103.

85. Gurwitz JHMD. Concomitant use of warfarin and cotrimoxazole or ciprofloxacin increased risk for admission for upper GI hemorrhage. ACP J Club. 153(1):JC1-13.

86. Hafner Jr. JW, Belknap SM, Squillante MD, Bucheit KA. Adverse drug events in emergency department patients. Ann Emerg Med. 2002;39(3):258-267.

87. Hirscb AC, Lundquist LM. Ciprofloxacin-induced hepatotoxicity resolved with levofloxacin: A case report and a review of the literature. Hosp Pharm. 2009;44(11):978-983.

88. Hofer-Dueckelmann C, Prinz E, Beindl W, et al. Adverse drug reactions (ADRs) associated with hospital admissions - Elderly female patients are at highest risk. Int J Clin Pharmacol Ther. 2011;49(10):577-586.

89. Holland EG, Degruy F V. Drug-induced disorders. Am Fam Physician. 1997;56(7):1781-1788.

90. Hsiao S-H, Chang C-M, Tsao C-J, Lee Y-YJ, Hsu M-Y, Wu T-J. Acute rhabdomyolysis associated with ofloxacin/levofloxacin therapy. Ann Pharmacother. 2005;39(1):146-149.

91. Huminer D, Cohen JD, Majadla R, Dux S. Hypersensitivity vasculitis due to ofloxacin. BMJ. 1989;299(6694):303.

92. Jha N, Bajracharya O, Namgyal T. Prevalence of adverse drug reactions with commonly prescribed drugs in different hospitals of Kathmandu valley. Kathmandu Univ Med J. 2007;5(20):504-510.

93. Johannes CB, Ziyadeh N, Seeger JD, Tucker E, Reiter C, Faich G. Incidence of allergic reactions associated with antibacterial use in a large, managed care organisation. Drug Saf. 2007;30(8):705-713.

94. Jones SC, Budnitz DS, Sorbello A, Mehta H. US-based emergency department visits for fluoroquinolone-associated hypersensitivity reactions. Pharmacoepidemiol Drug Saf. 2013;22(10):1099-1106.

95. Jones SC, Sorbello A, Boucher RM. Fluoroquinolone-associated myasthenia gravis exacerbation: evaluation of postmarketing reports from the US FDA adverse event reporting system and a literature review. Drug Saf. 2011;34(10):839-847.

96. Jose J, Rao PGM. Pattern of adverse drug reactions notified by spontaneous reporting in an Indian tertiary care teaching hospital. Pharmacol Res. 2006;54(3):226-233.

97. Joshua L, Devi P, Guido S. Adverse drug reactions in nephrology ward inpatients of a tertiary care hospital. Indian J Med Sci. 2007;61(10):562-569.

98. Kane-Gill S, Rea RS, Verrico MM, Weber RJ. Adverse-drug-event rates for high-cost and high-use drugs in the intensive care unit. Am J Heal Pharm. 2006;63(19):1876-1881.

99. Kanerva M, Ollgren J, Voipio T, Mentula S, Lyytikainen O. Regional differences in Clostridium difficile infections in relation to fluoroquinolone and proton pump inhibitor use, Finland, 2008-2011. Infect Dis (London, England). 2015;47(8):530-535.

100. Karakan Y, Akpinar A, Yildiz H, Aksoy H, Dikensoy O. A case of ciprofloxacin tablet aspiration. Tuberk Toraks. 2010;58(1):97-99.

101. Kelesidis T, Fleisher J, Tsiodras S. Anaphylactoid Reaction Considered Ciprofloxacin Related: A Case Report and Literature Review. Clin Ther. 2010;32(3):515-526.

102. Knorr JP, Moshfeghi M, Sokoloski MC. Ciprofloxacin-induced Q-T interval prolongation. Am J Heal Pharm. 2008;65(6):547-551.

103. Kocyigit I, Dortdudak S, Sipahioglu M, et al. Levofloxacin-induced delirium: Is it a dangerous drug in patients with renal dysfunction? Ren Fail. 2012;34(5):634-636.

104. Kranthi K, Jamuna Rani R, Sathyanarayanan V. Evaluation of ADR - A prospective analysis in a tertiary care teaching hospital. Int J Pharma Bio Sci. 2016;7(1):P56-P61.

105. Kurti Z, Lovasz BD, Mandel MD, et al. Burden of Clostridium difficile infection between 2010 and 2013: Trends and outcomes from an academic center in Eastern Europe. World J Gastroenterol. 2015;21(21):6728-6735.

106. Kusowska J. Cost-effectiveness analysis of therapy with standard antibiotics versus fluoroquinolone at Clinical Unit of diabetology, Medical University of Łódź, Poland. New Med. 2005;8(1).

107. Landen H, Bauer T. Efficacy, onset of action and tolerability of moxifloxacin in patients with community-acquired pneumonia: Results of a postmarketing surveillance study. Clin Drug Investig. 2001;21(12):801-811.

108. Lapi F, Wilchesky M, Kezouh A, Benisty JI, Ernst P, Suissa S. Fluoroquinolones and the risk of serious arrhythmia: a population-based study. Clin Infect Dis. 2012;55(11):1457-1465.

109. Lau I, Kirkwood A. Measuring adverse drug events on hospital medicine units with the Institute for Healthcare Improvement trigger tool: A chart review. Can J Hosp Pharm. 2014;67(6):423-428.

110. LeBlanc L, Pepin J, Toulouse K, et al. Fluoroquinolones and risk for methicillin-resistant Staphylococcus aureus, Canada. Emerg Infect Dis. 2006;12(9):1398-1405.

111. Lee C-C, Lee M-TG, Chen Y-S, et al. Risk of Aortic Dissection and Aortic Aneurysm in Patients Taking Oral Fluoroquinolone. JAMA Intern Med. 2015;175(11):1839-1847.

112. Leone R, Venegoni M, Motola D, et al. Adverse drug reactions related to the use of fluoroquinolone antimicrobials: An analysis of spontaneous reports and fluoroquinolone consumption data from three Italian regions. Drug Saf. 2003;26(2):109-120.

113. Letrilliart L, Hanslik T, Biour M, Fagot J-P, Guiguet M, Flahault A. Postdischarge adverse drug reactions in primary care originating from hospital care in France: A nationwide prospective study. Drug Saf. 2001;24(10):781-792.

114. Lim S, Alam MG. Ciprofloxacin-induced acute interstitial nephritis and autoimmune hemolytic anemia. Ren Fail. 2003;25(4):647-651.

115. Macy E, Contreras R. Health care use and serious infection prevalence associated with penicillin “allergy” in hospitalized patients: A cohort study. J Allergy Clin Immunol. 2014;133(3):790-796.

116. Makaryus AN, Byrns K, Makaryus MN, Natarajan U, Singer C, Goldner B. Effect of ciprofloxacin and levofloxacin on the QT interval: Is this a significant “clinical” event? South Med J. 2006;99(1):52-56.

117. Malladi SS, Liew EKS, Ng XT, Tan RKS. Ciprofloxacin eye drops-induced subtherapeutic serum phenytoin levels resulting in breakthrough seizures. Singapore Med J. 2014;55(7):e114-5.

118. Mandavia DR, Virpariya MM, Patel TK, Tripathi CB. Moxifloxacin-induced hypoglycemia in a non-diabetic patient. Curr Drug Saf. 2012;7(2):183-185.

119. Martin SJ, Jung R, Garvin CG. A risk-benefit assessment of levofloxacin in respiratory, skin and skin structure, and urinary tract infections. Drug Saf. 2001;24(3):199-222.

120. Mathis AS, Chan V, Gryszkiewicz M, Adamson RT, Friedman GS. Levofloxacin-associated Achilles tendon rupture. Ann Pharmacother. 2003;37(7-8):1014-1017.

121. McCusker ME, Harris AD, Perencevich E, Roghmann M-C. Fluoroquinolone use and Clostridium difficile-associated diarrhea. Emerg Infect Dis. 2003;9(6):730-733.

122. McDermott JL, Gideonse N, Campbell JW. Acute delirium associated with ciprofloxacin administration in a hospitalized elderly patient. J Am Geriatr Soc. 1991;39(9):909-910.

123. Mehlhorn AJ, Brown DA, Mehhorn AJ, Brown DA. Safety concerns with Fluoroquinolones. Ann Pharmacother. 2007;41(11):1859-1866.

124. Meropol SB, Chan KA, Chen Z, et al. Adverse events associated with prolonged antibiotic use. Pharmacoepidemiol Drug Saf. 2008;17(5):523-532.

125. Minson Q, Mok S. Relationship between antibiotic exposure and subsequent Clostridium difficile-associated diarrhea. Hosp Pharm. 2007;42(5):430-434.

126. Misra UK, Kalita J, Chandra S, Nair PP. Association of antibiotics with status epilepticus. Neurol Sci. 2013;34(3):327-331.

127. Mittmann N, Knowles SR, Gomez M, Fish JS, Cartotto R, Shear NH. Evaluation of the extent of under-reporting of serious adverse drug reactions: The case of toxic epidermal necrolysis. Drug Saf. 2004;27(7):477-487.

128. Montoya M, Detorres O. Antimicrobial selection and its impact on the incidence of Clostridium difficile-associated diarrhea. J Pharm Pract. 2013;26(5):483-487.

129. Moore GC, Redfern J, Shiach CR, Webb K, Jones AM. Coagulopathy in two patients with cystic fibrosis treated with ciprofloxacin. J Cyst Fibros. 2007;6(3):209-211.

130. Moshfeghi M, Mandler HD. Ciprofloxacin-induced toxic epidermal necrolysis. Ann Pharmacother. 1993;27(12):1467-1469.

131. Naveen KN, Pai V V, Rai V, Athanikar SB. Retrospective analysis of Steven Johnson syndrome and toxic epidermal necrolysis over a period of 5 years from northern Karnataka, India. Indian J Pharmacol. 2013;45(1):80-82.

132. Niedrig D, Maechler S, Hoppe L, Corti N, Kovari H, Russmann S. Drug safety of macrolide and quinolone antibiotics in a tertiary care hospital: administration of interacting co-medication and QT prolongation. Eur J Clin Pharmacol. 2016;72(7):859-867.

133. Nightingale CH, Quintiliani R. Cost of oral antibiotic therapy. Pharmacotherapy. 1997;17(2):302-307.

134. Oh YR, Carr-Lopez SM, Probasco JM, Crawley PG. Levofloxacin-induced autoimmune hemolytic anemia. Ann Pharmacother. 2003;37(7-8):1010-1013.

135. Olsen MA, Yan Y, Reske KA, Zilberberg M, Dubberke ER. Impact of Clostridium difficile recurrence on hospital readmissions. Am J Infect Control. 2015;43(4):318-322.

136. Owens RCJ, Ambrose PG. Antimicrobial safety: focus on fluoroquinolones. Clin Infect Dis. 2005;41 Suppl 2:S144-57.

137. Paladino JA Backes JM, Gelber JA, Serrianne DJ, Cumbo TJ, Schentag JJ SHE, Paladino JA, Sperry HE, et al. Clinical and economic evaluation of oral ciprofloxacin after an abbreviated course of intravenous antibiotics. Am J Med. 1991;91(5):462.

138. Paladino JA. Pharmacoeconomic comparison of sequential IV/oral ciprofloxacin versus ceftazidime in the treatment of nosocomial pneumonia. Can J Hosp Pharm. 1995;48(5):276-283.

139. Panicker GK Kadam P, Badilini F, Damle A, Kothari S KDR, Panicker GK, Karnad DR, et al. Detecting moxifloxacin-induced QTc prolongation in thorough QT and early clinical phase studies using a highly automated ECG analysis approach. Br J Pharmacol. 2016;173(8):1373.

140. Park MY, Kim EY, Lee YH, et al. Analysis of relationship between levofloxacin and corrected QT prolongation using a clinical data warehouse. Healthc Inform Res. 2011;17(1):58-66.

141. Patel J, Desai M, Mishra V, Shah S. Evaluation of ophthalmic adverse drug reactions at a tertiary-care hospital. Drugs Ther Perspect. 2015;31(12):448-451.

142. Patel NS. Fluoroquinolone use is the predominant risk factor for the development of a new strain of Clostridium difficile-associated disease. BJU Int. 2007;99(6):1333-1334.

143. Patel N, Desai S. Profile of adverse drug reactions in patients admitted to general surgical wards of a rural tertiary-care hospital in India. Drugs Ther Perspect. 2015;31(11):402-406.

144. Patel PD, Afshar H, Birnbaum Y. Levofloxacin-induced torsades de pointes. Texas Hear Inst J. 2010;37(2):216-217.

145. Patel TK, Barvaliya MJ, Sharma D, Tripathi C. A systematic review of the drug-induced Stevens-Johnson syndrome and toxic epidermal necrolysis in Indian population. Indian J Dermatol Venereol Leprol. 2013;79(3):389-398.

146. Patro N, Panda M, Jena M, Mishra S. Multifocal fixed drug eruptions: A case series. Int J Pharm Sci Rev Res. 2013;23(1):63-66.

147. Pépin J, Saheb N, Coulombe M-A, et al. Emergence of fluoroquinolones as the predominant risk factor for Clostridium difficile-associated diarrhea: A cohort study during an epidemic in Quebec. Clin Infect Dis. 2005;41(9):1254-1260.

148. Petitjeans F, Nadaud J, Perez JP, et al. A case of rhabdomyolysis with fatal outcome after a treatment with levofloxacin. Eur J Clin Pharmacol. 2003;59(10):779-780.

149. Ponnusankar S, Tejaswini M, Chaitanya M. Assessment of Adverse Drug Reactions Based on Spontaneous Signals at Secondary Care Public Hospital. INDIAN J Pharm Sci. 2015;77(4):490-493.

150. Raguideau F, Lemaitre M, Dray-Spira R, Zureik M. Association Between Oral Fluoroquinolone Use and Retinal Detachment. JAMA Ophthalmol. 2016;134(4):415-421.

151. Rahmati-Roodsari M, Shadnia S, Abdollahi M. Drug-induced skin events in hospitalized patients in Tehran, Iran: A 6-year case series study. Arch Med Sci. 2009;5(1):91-96.

152. Raj V, Murthy TVSP. Levofloxacin induced delirium with psychotic features in a young patient. Med J Armed Forces India. 2013;69(4):404-405.

153. Rao GG, Rao CSM, Starke I. Clostridium difficile-associated diarrhoea in patients with community-acquired lower respiratory infection being treated with levofloxacin compared with beta-lactam-based therapy. J Antimicrob Chemother. 2003;51(3):697-701.

154. Raut A, Pawar A, Pankaj M, Srivastava P, Mishra A. Clinical pattern and severity of cutaneous adverse drug reactions. Int J Pharm Pharm Sci. 2013;5(SUPPL. 2):612-616.

155. Rehan HS, Chopra D, Sah RK, Mishra R. Adverse drug reactions: Trends in a tertiary care hospital. Curr Drug Saf. 2012;7(5):384-388.

156. Reis AMM, Cassiani SHDB. Adverse drug events in an intensive care unit of a university hospital. Eur J Clin Pharmacol. 2011;67(6):625-632.

157. Reti IM, Davydow DS. Electroconvulsive therapy and antibiotics: A case report. J ECT. 2007;23(4):289-290.

158. Richa, Tandon VR, Sharma S, Khajuria V, Mahajan V, Gillani Z. Adverse drug reactions profile of antimicrobials: A 3-year experience, from a tertiary care teaching hospital of India. INDIAN J Med Microbiol. 2015;33(3):393-400.

159. Richerson MA, Ambrose PG, Quintiliani R, Bui KQ, Nightingale CH. Pharmacoeconomic evaluation of alternative antibiotic regimens in hospitalized patients with community-acquired pneumonia. Infect Dis Clin Pract. 1998;7(5):227-233.

160. Sahin MT, Ozturkcan S, Inanir I, Filiz EE. Norfloxacin-induced toxic epidermal necrolysis. Ann Pharmacother. 2005;39(4):768-770.

161. Salloum R, Liu CY, Weise AM. Possible case of levofloxacin-induced thrombocytopenia. Am J Health Syst Pharm. 2011;68(1):43-46.

162. Samoy LJ, Zed PJ, Wilbur K, Balen RM, Abu-Laban RB, Roberts M. Drug-related hospitalizations in a tertiary care internal medicine service of a Canadian hospital: A prospective study. Pharmacotherapy. 2006;26(11):1578-1586.

163. Sawicki J, Ellis AK. Stevens-Johnson syndrome: A review of 14 adult cases with one fatal outcome. Ann Allergy, Asthma Immunol. 2013;110(3):207-209e1.

164. Schacht P, Arcieri G, Hullmann R. Safety of oral ciprofloxacin. An update based on clinical trial results. Am J Med. 1989;87(5A):98S-102S.

165. Schindler M, Bernard L, Belaieff W, et al. Epidemiology of adverse events and Clostridium difficile-associated diarrhea during long-term antibiotic therapy for osteoarticular infections. J Infect. 2013;67(5):433-438.

166. Seeger JD, West WA, Fife D, Noel GJ, Johnson LN, Walker AM. Achilles tendon rupture and its association with fluoroquinolone antibiotics and other potential risk factors in a managed care population. Pharmacoepidemiol Drug Saf. 2006;15(11):784-792.

167. Shamna M, Dilip C, Ajmal M, et al. A prospective study on Adverse Drug Reactions of antibiotics in a tertiary care hospital. SAUDI Pharm J. 2014;22(4):303-308.

168. Shehab N, Patel PR, Srinivasan A, Budnitz DS. Emergency department visits for antibiotic-associated adverse events. Clin Infect Dis. 2008;47(6):735-743.

169. Shortt P, Wilson R, Erskine I. Tendinitis: the Achilles heel of quinolones! Emerg Med J. 2006;23(12):e63.

170. Shuster J. Ciprofloxacin-induced immunoglobulin a disease; Palonosetron-induced anaphylaxis; Guillain-Barré Syndrome following H1N1 immunization; Acute profound thrombocytopenia following eptifibatide administration; Clozapine-associated cerebral venous thrombosis. Hosp Pharm. 2010;45(9):680-684.

171. Shuster J. Rhabdomyolysis associated with fluoroquinolones - Rhabdomyolysis and myopathy with combined therapy for hyperlipidemia. Incidence of rhabdomyolysis in hospitalized patients and conflict of interest controversy: Abdominal cramping associated with prostagla. Hosp Pharm. 2005;40(3):214-217.

172. Slobodin G, Elias N, Zaygraikin N, et al. Levofloxacin-induced delirium. Neurol Sci. 2009;30(2):159-161.

173. Smythe MA, Cappelletty DM. Anaphylactoid reaction to levofloxacin. Pharmacotherapy. 2000;20(12):1520-1523.

174. Son CH, Kim HI, Kim KN, et al. Moxifloxacin-associated drug hypersensitivity syndrome with drug-induced hypersensitivity pneumonitis. J Investig Allergol Clin Immunol. 2008;18(1):72-73.

175. Stancampiano FF, Palmer WC, Getz TW, et al. Rare Incidence of Ventricular Tachycardia and Torsades de Pointes in Hospitalized Patients With Prolonged QT Who Later Received Levofloxacin: A Retrospective Study. Mayo Clin Proc. 2015;90(5):606-612.

176. Starr JA, Ragucci KR. Thrombocytopenia associated with intravenous ciprofloxacin. Pharmacotherapy. 2005;25(7):1030-1034.

177. Steinert T, Studemund H. Acute delusional parasitosis under treatment with ciprofloxacin: A case report. Pharmacopsychiatry. 2006;39(4):159-160.

178. Sun H-Y, Chen Y-C, Wang Y-W, Gau C-S, Chang S-C. A prospective study of antimicrobial-related adverse drug reactions in hospitalized patients. J Microbiol Immunol Infect. 2008;41(2):151-159.

179. Sung HY, Kim JI, Lee HJ, et al. Acute pancreatitis secondary to ciprofloxacin therapy in patients with infectious colitis. Gut Liver. 2014;8(3):265-270.

180. Tachi T, Teramachi H, Asano S, et al. Impact of levofloxacin dose adjustments by dispensing pharmacists on adverse reactions and costs in the treatment of elderly patients. Pharmazie. 2013;68(12):977-982.

181. Tohyama M, Arakaki N, Tamaki K, Shimoji T. [A case of drug-induced pneumonitis due to levofloxacin and kampo medicine]. Nihon Kokyuki Gakkai Zasshi. 2006;44(12):951-956.

182. Tomé AM, Filipe A. Quinolones: Review of psychiatric and neurological adverse reactions. Drug Saf. 2011;34(6):465-488.

183. Traynor K. Study examines adverse-drug-event costs for antimicrobials. Am J Heal Pharm. 2006;63(3):206-208.

184. Trifirò G, Calogero G, Ippolito FM, et al. Adverse drug events in emergency department population: A prospective Italian study. Pharmacoepidemiol Drug Saf. 2005;14(5):333-340.

185. Tripathy S, Adhya A. Ofloxacin induced leucopenia in complicated falciparum malaria: A case report. Cases J. 2009;2(6).

186. Trubiano JA, Aung AK, Nguyen M, et al. A Comparative Analysis Between Antibiotic- and Nonantibiotic-Associated Delayed Cutaneous Adverse Drug Reactions. J ALLERGY Clin Immunol Pract. 2016;4(6):1187-1193.

187. Tsai L-H, Weng Y-M, Lin C-C, Kuo C-W, Chen J-C. Risk screening for long QT prior to prescribing levofloxacin. Am J Emerg Med. 2014;32(9).

188. Tuccori M, Guidi B, Carulli G, Blandizzi C, Del Tacca M, Di Paolo M. Severe thrombocytopenia and haemolytic anaemia associated with ciprofloxacin: a case report with fatal outcome. Platelets. 2008;19(5):384-387.

189. Turk BG, Gunaydin A, Ertam I, Ozturk G. Adverse cutaneous drug reactions among hospitalized patients: Five year surveillance. Cutan Ocul Toxicol. 2013;32(1):41-45.

190. Uchit GP, Shrivastava MP, Badar VA, Navale SB, Mayabhate MM. Adverse drug reactions to antimicrobial agents in a tertiary care hospital in Nagpur. J Indian Med Assoc. 2012;110(4):224-227.

191. Walton GD, Hon JK, Mulpur TG. Ofloxacin-induced seizure. Ann Pharmacother. 1997;31(12):1475-1477.

192. Van Bambeke F, Tulkens PM. Safety profile of the respiratory fluoroquinolone moxifloxacin: Comparison with other fluoroquinolones and other antibacterial classes. Drug Saf. 2009;32(5):359-378.

193. Van Berkel MA, Twilla JD, England BS. Emergency Department Management of a Myasthenia Gravis Patient with Community-Acquired Pneumonia: Does Initial Antibiotic Choice Lead to Cure or Crisis? J Emerg Med. 2016;50(2):281-285.

194. Van Der Linden PD, Stricker BHC, Leufkens HGM, Herings RMC, Nab HW, Simonian S. Fluoroquinolone use and the change in incidence of tendon ruptures in the Netherlands. Pharm World Sci. 2001;23(3):89-92.

195. van der Linden PD, van Puijenbroek EP, Feenstra J, et al. Tendon disorders attributed to fluoroquinolones: a study on 42 spontaneous reports in the period 1988 to 1998. Arthritis Rheum. 2001;45(3):235-239.

196. VanderBeek BL. Oral fluoroquinolones, retinal detachments, and claims database studies. JAMA Ophthalmol. 2016;134(4):422-423.

197. Wang F, Li Y, Mo Y, Shen C, Yang L, Zhang X. Cutaneous adverse drug reactions: an 8-year retrospective study on hospitalized patients in Southern China. Indian J Dermatol Venereol Leprol. 2012;78(4):488-490.

198. Wang S, Xie Y, Jiang B, et al. [Fluoroquinolone associated myasthenia gravis exacerbation: clinical analysis of 9 cases]. Zhonghua Yi Xue Za Zhi. 2013;93(17):1283-1286.

199. Weingart SN, Simchowitz B, Padolsky H, et al. An empirical model to estimate the potential impact of medication safety alerts on patient safety, health care utilization, and cost in ambulatory care. Arch Intern Med. 2009;169(16):1465-1473.

200. Weiss K. Clostridium difficile and fluoroquinolones: is there a link? Int J Antimicrob Agents. 2009;33(SUPPL. 1):S29-S32.

201. Vesteinsdottir I, Gudlaugsdottir S, Einarsdottir R, Kalaitzakis E, Sigurdardottir O, Bjornsson ES. Risk factors for Clostridium difficile toxin-positive diarrhea: a population-based prospective case-control study. Eur J Clin Microbiol Infect Dis. 2012;31(10):2601-2610.

202. Villeneuve JP, Davies C, Cote J. Suspected ciprofloxacin-induced hepatotoxicity. Ann Pharmacother. 1995;29(3):257-259.

203. WIN A, EVERS ML, CHMEL H. STEVENS-JOHNSON SYNDROME PRESUMABLY INDUCED BY CIPROFLOXACIN. Int J Dermatol. 1994;33(7):512-514.

204. Vodovar D, LeBeller C, Megarbane B, Lillo-Le-Louet A, Hanslik T. Drug Fever: a descriptive cohort study from the French national pharmacovigilance database. Drug Saf. 2012;35(9):759-767.

205. Wu B-Q, Pradhan YC, Zhang T-T, Huang J, Zhu J-X. Comparing therapeutic and adverse effects of moxifloxacin and levofloxacin in treatment of community acquired pneumonia: A meta-analysis of randomized controlled trials. AFRICAN J Microbiol Res. 2012;6(9):1893-1901.

206. Yam FK, Eraly SA. Syndrome of inappropriate antidiuretic hormone associated with moxifloxacin. Am J Health Syst Pharm. 2012;69(3):217-220.

207. Yasuda H, Yoshida A, Masuda Y, Fukayama M, Kita Y, Inamatsu T. [Levofloxacin-induced neurological adverse effects such as convulsion, involuntary movement (tremor, myoclonus and chorea like), visual hallucination in two elderly patients]. Nihon Ronen Igakkai Zasshi. 1999;36(3):213-217.

208. Yee JL, Hasson NK, Schreiber DH. Drug-related emergency department visits in an elderly veteran population. Ann Pharmacother. 2005;39(12):1990-1995.

209. Yildirim P. Association Patterns in Open Data to Explore Ciprofloxacin Adverse Events. Appl Clin Inform. 2015;6(4):728-747.

210. Yoon YK, Kim ES, Hur J, et al. Oral antimicrobial therapy: Efficacy and safety for methicillin-resistant Staphylococcus aureus infections and its impact on the length of hospital stay. Infect Chemother. 2014;46(3):172-181.

211. Zilberberg MD, Reske K, Olsen M, Yan Y, Dubberke ER. Risk factors for recurrent Clostridium difficile infection (CDI) hospitalization among hospitalized patients with an initial CDI episode: a retrospective cohort study. BMC Infect Dis. 2014;14:306.

212. Fluoroquinolones: Psychiatric adverse effects. Prescrire Int. 2008;17(93):20.
