# Supplementary material for: Fluoroquinolone-related adverse events resulting in health service use and costs: A systematic review
Source: PLoS One. 2019 Apr 26;14(4):e0216029. doi: 10.1371/journal.pone.0216029 (PMC6485715; doi:10.1371/journal.pone.0216029)
Supplement: S1 Checklist — (PDF) [file pone.0216029.s004.pdf]

S4 Checklist. PRISMA Checklist.

Section/topic	#	Checklist item	Reported on page #
**TITLE**			
Title	1	"Fluoroquinolone-related Adverse Events Resulting in Health Service Use and Costs: A Systematic Review"	Title
**ABSTRACT**			
Structured summary	2	"Background and Objectives: Adverse events (AEs) associated with the use of fluoroquinolone antimicrobials include *Clostridium difficile* associated diarrhea (CDAD), liver injury and seizures. Yet, the economic impact of these AEs is seldom acknowledged. The aim of this review was to identify health service use and subsequent costs associated with ciprofloxacin, levofloxacin, moxifloxacin, norfloxacin and ofloxacin -related AEs.<br>Methods: A literature search covering Medline, SCOPUS, Cinahl, Web of Science and Cochrane Library was performed in April 2017. Two independent reviewers systematically extracted the data and assessed the quality of the included studies. All costs were converted to 2016 euro in order to improve comparability.<br>Results: Of the 5,687 references found in the literature search, 19 observational studies, of which five were case-controlled, fulfilled the inclusion criteria. Hospitalization was an AE-related health service use outcome in 17 studies. Length of hospital stay associated with AEs varied between <5 and 45 days. The estimated cost of an AE episode ranged between 140 and 18,252 €. CDAD was associated with the longest stays in hospital. Ten studies reported AE-related length of stays and five evaluated costs associated with AEs. Due to the lack of published literature, health service use and costs associated with many high-risk FQ-related AEs could not be evaluated.<br>Conclusions: Because of the wide clinical use of fluoroquinolones, in particular serious fluoroquinolone-related AEs can have substantial economic implications, in addition to imposing potentially devastating health complications for patients. Further measures are required to prevent and reduce health service use and costs associated with fluoroquinolone-related AEs. Equally, better-quality reporting and additional published data on health service use and costs associated with AEs are needed."	Abstract
**INTRODUCTION**			
Rationale	3	"The economic burden of AEs is substantial and in direct relation to current increasing drug utilization. According to previous research, the annual cost of AEs in the U.S. may be as high as 22.9 billion euros [12]. In Europe AEs are considered to contribute to 3.6 percent of hospital admissions, have an impact on 10 percent of inpatients during their hospital admission and are responsible for almost 0.5 percent of inpatient deaths. [13] AEs thus clearly constitute a major clinical issue. Prescribing a drug is always a conflict of benefits set against harms decision, weighing the risk of morbidity and even mortality from the disease against similar effects from AEs and added health care costs. Unfortunately, a thorough understanding of the significance of AEs and the benefit-risk-ratio of drug treatments can only be acquired through long-term clinical use after marketing authorization and subsequent research. Health service use and costs specifically associated with FQ-related AEs have not been evaluated previously."	Introduction

Objectives	4	"The aim of our study was to identify health service use and costs associated with ciprofloxacin, levofloxacin, moxifloxacin, norfloxacin and ofloxacin -related AEs."	Introduction
**METHODS**			
Protocol and registration	5	-	Not applicable
Eligibility criteria	6	"References identified in the literature search were imported to reference management software (Mendeley) and duplicates were removed. Only references that met previously fixed PICOS (patients, intervention, control, outcome, setting) [16] criteria, were included in the review. There were no limitations concerning publication year. The PICOS framework is depicted in Table 1."	Methods (Study Selection)<br>Table 1
Information sources	7	"A systematic literature search was performed in April 2017 covering Medline, SCOPUS, CINAHL, Web of Science and Cochrane Library. A library information specialist was consulted in forming the search strategies, which consisted of search terms relating to fluoroquinolones, adverse events, health service use and costs. The Web of Science -database search included several conference papers, which could be used to find unpublished literature and reduce publication bias. Finally, literature references of the included articles were sourced to identify potentially relevant articles."	Methods (Literature Search)
Search	8	"The search strategy for Medline can be found in S2."	S2
Study selection	9	"Both reviewers (LS, KV) individually screened the articles based on title and excluded distinctly irrelevant references. A third author (MB) was available to resolve possible discrepancies. The remaining articles were screened based on abstracts and full texts."	Methods (Study Selection)
Data collection process	10	"The data of the included articles was extracted into two spread sheets (Microsoft Excel). The usefulness of the tables was tested with a total of eight articles, after which minor adjustments were made regarding the reporting of fatalities. Both reviewers (LS, KV) filled in the tables independently."	Methods (Data Collection)
Data items	11	"The first table contains characteristics of the included studies, such as authors, publication years, aims, patient details, study designs, durations, follow-ups, funding details and publications. The second table summarizes results covering specifics of the fluoroquinolone associated with the adverse event, adverse event types, health service use, length of hospital stay, AE costs and possible fatalities."	Methods (Data Collection)
Risk of bias in individual studies	12	"The quality of the included studies was assessed according to the STROBE checklist for observational studies.[19] The studies were awarded scores, which are presented in percentages. Two reviewers (LK, KV) assessed the quality of the included studies independently."	Methods (Data Collection)
Summary measures	13	"In order to improve comparability, all the reported costs were converted to euro by using the exchange rate of the European Central Bank and adjusted to the price level of the year 2016 using the value of money index of Statistics Finland."	Methods (Quality Assessment)
Synthesis of results	14	-	Not applicable

Section/topic	#	Checklist item	Reported on page #
Risk of bias across studies	15	-	Not applicable
Additional analyses	16	-	Not applicable
**RESULTS**			
Study selection	17	"In all, 4,454 unique references were identified in the literature search (Figure 1). Screening based on titles excluded 4,217 references. Two hundred and twenty full-text articles did either not meet the inclusion criteria (n=208 studies), were found to be duplicates (n=8) or lacked an English language full-text (n=4). After two additional studies were found in literature references, a total of 19 studies were included in this systematic review."	Results (Fig 1.)
Study characteristics	18	"Of the 19 included observational studies ([20]-[31]), five were case-controlled ([20][21][22][23][24]). The studies were published between 2002 and 2017. There were substantial differences in study duration, the length varied from 4 weeks to 22 years. The total sample size of the included studies comprised 1,752,544 patients. During the study periods, 33,477 AEs that were identified as FQ-related occurred. The studies included 22,704 AEs associated with levofloxacin, 339 with ciprofloxacin, two with norfloxacin, three with ofloxacin and 168 with moxifloxacin. In total, 10,773 AEs were associated with an unspecified FQ. A total of 26,893 (80%) were identified from one study[25]. The average age of all total sample was 60,8 years and 50,71% were men. Only one study explicitly involved a cohort of patients with comorbidities (diabetes).[26] The characteristics of the included studies are summarized in Table 2."	Results (Table 2)
Risk of bias within studies	19	"The results of the quality assessment are illustrated in Figure 2. The included studies scored an average 19.74 and median 20 (range 10 and 27) points out of 34 total points. The weighted average rating was 65% (range 36-84%). Although the scores are relatively high, some inadequacies were apparent in reporting. Only six studies described efforts to address potential sources of bias ([20]-[22][24][26][27])."	Results (The Quality of the Included Studies, Fig 2.)
Results of individual studies	20	"Health service use and and costs associated with FQ-related AEs are depicted in table 3."	Results (Table 3)
Synthesis of results	21	-	Not applicable
Risk of bias across studies	22	-	Not applicable
Additional analysis	23	-	Not applicable
**DISCUSSION**			

Summary of evidence	24	"In this systematic review, hospitalizations and ED visits were the main health service use outcomes associated with AEs. Outpatient visits to primary care facilities were not reported in the included studies, although it is likely that most AEs are diagnosed and treated in primary care, if recognized as FQ-related at all. According to prior research by Magdelijns et.al., hospitalizations, specifically long stays in hospital, are the leading cost drivers in health service use. Hospitalizations were estimated to cause approximately 77% of direct health care costs associated with AEs in the Netherlands[41]. Reported FQ-related AE-costs varied between 140 and 18,252€ per AE episode. CDAD was associated with the largest amount of health service use, longest stays in hospital and, thus, the highest reported costs of AEs considered. Mean CDAD-related length of stays were up to 45 days. Since the emergence of the epidemic *Clostridium difficile* ribotype 027 clone, CDAD has become more prevalent, severe and more difficult to treat, due to resistance to many antimicrobial agents[42]."	Discussion
Limitations	25	"Limitations of this systematic review include confining the literature search to full English language texts. However, the risk of lost key findings is minor due to the paucity of non-English texts excluded from the review. In addition, we excluded studies with pediatric patients, though inclusion could have led to added information about health service use and costs. The use of FQs in children continues to be limited or restricted. Although studies have described the majority of FQ-related AEs in pediatric patients as temporary and reversible [43], real-world safety data continue to be scarce. We acknowledge that the use of STROBE checklist for observational studies is not recommended for assessing the methodological quality of studies. There is a distinct deficiency of reliable, comprehensive and validated tools for the quality assessment of observational studies. We did not exclude any studies due to poor quality and therefore using STROBE did not introduce bias into this systematic review. Additionally, there is a lack of guidelines and definitions regarding data quality, which is not addressed in quality assessments. This could potentially cause bias. The shortage of existing research relating to health service use and costs associated with FQ-related AEs and the incomplete nature of AEs considered in those that do report these, account for the largest limitation of this systematic review."	Discussion
Conclusions	26	"Because of the wide clinical use of FQs, in particular serious fluoroquinolone-related AEs can have substantial economic implications, in addition to imposing potentially long-lasting health complications for patients. Better-quality reporting and additional published data on health service use and costs associated with AEs are both necessary and overdue."	Conclusions
**FUNDING**			
Funding	27	The authors have no support or funding to disclose.	

From: Moher D, Liberati A, Tetzlaff J, Altman DG, The PRISMA Group (2009). Preferred Reporting Items for Systematic Reviews and Meta-Analyses: The PRISMA Statement. PLoS Med 6(7): e1000097. doi:10.1371/journal.pmed1000097 For more information, visit: www.prisma-statement.org.
